# DPF is a cell-density sensing factor, with cell-autonomous and non-autonomous functions during *Dictyostelium* growth and development

**DOI:** 10.1186/s12915-019-0714-9

**Published:** 2019-12-02

**Authors:** Netra Pal Meena, Pundrik Jaiswal, Fu-Sheng Chang, Joseph Brzostowski, Alan R. Kimmel

**Affiliations:** 1 Laboratory of Cellular and Developmental Biology, National Institute of Diabetes and Digestive and Kidney Diseases, The National Institutes of Health, Bethesda, MD 20892 USA; 2 Laboratory of Immunogenetics Twinbrook Imaging Facility, National Institute of Allergy and Infectious Diseases, The National Institutes of Health, Rockville, MD 20852 USA

**Keywords:** Signaling, Chemotaxis, Protein purification, MS/MS peptide sequencing, Ecto-domain shedding

## Abstract

**Background:**

Cellular functions can be regulated by cell-cell interactions that are influenced by extra-cellular, density-dependent signaling factors. *Dictyostelium* grow as individual cells in nutrient-rich sources, but, as nutrients become depleted, they initiate a multi-cell developmental program that is dependent upon a cell-density threshold. We hypothesized that novel secreted proteins may serve as density-sensing factors to promote multi-cell developmental fate decisions at a specific cell-density threshold, and use *Dictyostelium* in the identification of such a factor.

**Results:**

We show that multi-cell developmental aggregation in *Dictyostelium* is lost upon minimal (2-fold) reduction in local cell density. Remarkably, developmental aggregation response at non-permissive cell densities is rescued by addition of conditioned media from high-density, developmentally competent cells. Using rescued aggregation of low-density cells as an assay, we purified a single, 150-kDa extra-cellular protein with density aggregation activity. MS/MS peptide sequence analysis identified the gene sequence, and cells that overexpress the full-length protein accumulate higher levels of a development promoting factor (DPF) activity than parental cells, allowing cells to aggregate at lower cell densities; cells deficient for this *DPF* gene lack density-dependent developmental aggregation activity and require higher cell density for cell aggregation compared to WT. Density aggregation activity co-purifies with tagged versions of DPF and tag-affinity-purified DPF possesses density aggregation activity. In mixed development with WT, cells that overexpress DPF preferentially localize at centers for multi-cell aggregation and define cell-fate choice during cytodifferentiation. Finally, we show that DPF is synthesized as a larger precursor, single-pass transmembrane protein, with the p150 fragment released by proteolytic cleavage and ectodomain shedding. The TM/cytoplasmic domain of DPF possesses cell-autonomous activity for cell-substratum adhesion and for cellular growth.

**Conclusions:**

We have purified a novel secreted protein, DPF, that acts as a density-sensing factor for development and functions to define local collective thresholds for *Dictyostelium* development and to facilitate cell-cell communication and multi-cell formation. Regions of high DPF expression are enriched at centers for cell-cell signal-response, multi-cell formation, and cell-fate determination. Additionally, DPF has separate cell-autonomous functions for regulation of cellular adhesion and growth.

## Background

Cell-density sensing is broadly associated with response to signaling molecules that accumulate in the extracellular milieu in proportion to cellular mass. While perhaps often described as quorum sensing in the context of bacterial sociality and virulence [1], it is recognized that secreted factors in both prokaryotes and eukaryotes are sensed and function most effectively at threshold concentrations that directly reflect local cell density [2]. As example, where it is critical to accumulate sufficient cell mass to ensure productive organ development, cell proliferation may proceed at the expense of developmental processes [3–5]. Thereby, the secretion and accumulation of dependent concentrations of specific molecules can be a read-out for effective cell density. Other secreted, regulatory factors modulate action at differential signal strengths, thus directing distinct distal/proximal events from centers of the dispersing signal origin [6–8]. Changing concentrations relative to distance thereby provide an effective parallel for monitoring the local collective cellular environmental.

Many extracellular signaling molecules have been described, and their functions quite varied, underscoring importance to understand the nature of factors that affect a fundamental switch in developmental cell fate. *Dictyostelium* are social amoeboid eukaryotes with growth and developmental characteristics that make them highly suited to explore cell density-dependent accumulation of such extracellular signaling molecules.

*Dictyostelium* grow in the wild as individual cells, engulfing bacteria as a food source [9–11]. If bacteria are fully cleared within an area of an expanding population of *Dictyostelium*, the cells become starved for nutrients and enter a phase for multi-cell aggregate formation to maximize survival by differentiation, development, movement, and finally dispersal to regions with new, abundant nutrient sources. Development and survival are compromised at cell numbers below an optimized target, and the size-area of aggregation territories, as reflective of participating cell numbers, is highly regulated [12–16].

Within a nutrient depleted area, *Dictyostelium* cells establish signaling centers at stochastic intervals for production and secretion of the chemoattractant cAMP in temporal waves [10, 17]. Proximal cells respond by movement inward toward these centers of wave production and by relay outward of cAMP to recruit additional more distal cells. Secreted waves of cAMP also synchronize cAMP timing in all cells within the defining territory, to ensure a single dominating cAMP signaling center to collect cells for aggregate formation [18, 19]. Mutants or pharmaceuticals that enhance or suppress cAMP signaling, respectively, increase or decrease numbers of signaling centers and reciprocally territory size [12, 20–23].

*Dictyostelium* has been an ideal system for identification of extracellular proteins that regulate proliferation and growth or development and fate choice, and molecules, in addition to cAMP, can be secreted by *Dictyostelium* to allow cells to assess their near cell density to promote aggregation for optimal development and survival [12–16]. Chalones are secreted proteins that limit rates of cell proliferation, to control cell numbers in developing tissues. The AprA-CfaD complex in *Dictyostelium* exhibits chalone-like negative feedback control that limits cell proliferation [24, 25], whereas other secreted factors appear to completely block cell division [26]. PSF, the pre-starvation factor, accumulates in the media of cells entering stationary growth, but prior to the initiation of development [27, 28]. PSF primes cells for developmental response by inducing low expression of genes that will be required for starvation-induced cAMP response and control of early development. CF (Countin Factor) proteins CtnA, CtnB, etc., inversely control group size of developing *Dictyostelium* [29–31], whereas CMF, conditioned media factor, will promote *Dictyostelium* cytodifferentiation under conditions of extreme cell dilution and in the absence of cell-cell contact [32].

We were interested to identify secreted molecules that regulate cell density-dependent developmental processes. We show that *Dictyostelium* at low cell-density conditions are unable to form multi-cell developmental aggregates upon nutrient withdrawal; however, addition of media from developmentally competent cells allowed aggregation at these non-permissive cell densities. We identified a density aggregation activity as a 150-kDa protein (p150) that is secreted by ectodomain shedding of a single-pass transmembrane precursor. Gain-of-function studies show that the density-dependent aggregation activity affinity purifies with p150 and that cells overexpressing this p150 development promoting factor (DPF) aggregate at non-permissive densities, below that required by parental cells. In contrast, cells lacking the gene for *DPF* are unable to aggregate at normal cell densities. We additionally show, using mixed development studies, that cells overexpressing DPF preferentially localize at centers for aggregate formation for cAMP signaling and cell-fate determination, relative to WT. Finally, DPF is synthesized as a precursor, single-pass transmembrane protein, which is processed by proteolytic cleavage and ectodomain shedding. The active 150-kDa N-terminus is released, while the TM/cytoplasmic domain remains cell-anchored and possesses cell-autonomous functions for cell-substratum adhesion and for cellular growth.

## Results

### *Dictyostelium* secrete a factor that modulates cell-density-dependent, developmental aggregation

**Fig. 1:**
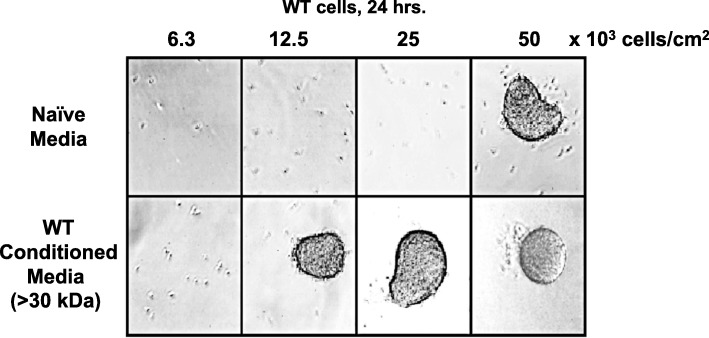
Conditioned media promotes aggregation at low cell density. Log phase growing WT cells were adhered in a 12-well plate under DB starvation buffer at indicated cell densities for 24 h, using either fresh, naïve DB media, or cell-free, > 30-kDa conditioned media from WT cells starved in DB for 18 h (see also Additional file 1: Figure S1A,B)

When deprived of nutrients, *Dictyostelium* initiate a developmental program leading to multi-cell aggregation [9–11]. Cells plated on a solid substrate in non-nutrient media secrete oscillating, nM waves of cAMP, which is the developmental chemoattractant that defines centers for multi-cell recruitment and aggregate formation [10, 12, 17, 20, 22]. However, the response processes that collectively mobilize cells at aggregation centers are highly dependent on cell density [12–16, 21, 23]; a two-fold reduction in cell density can be sufficient to strongly suppress aggregation (see Fig. 1; Additional file 1: Figure S1A,B,C). Certainly, several groups have described various secreted proteins in *Dictyostelium* that show increased accumulation in parallel to cell growth and serve as effective density sensing factors [12]. We were interested to identify additional secreted factors that regulate *Dictyostelium* development in a cell-density-dependent manner. We approached this, using aggregation as a read-out for developmental progression and by developing starved cells under-buffer in microtitre plastic dish wells, where added factors would not be diluted by absorption into supporting matrices, such as agar or filter pads.

WT *Dictyostelium* were washed from growth media and allowed to starve overnight in developmental buffer (DB), at 1–2 × 10^7^ cells/ml in shaking culture. The conditioned media from the starved cells were collected and a cell-free, > 30 kDa fraction prepared by filter centrifugation; we chose a size fraction cut-off to remove effects of secreted cAMP and other small molecules. Growth-phase WT cells were then washed and resuspended in either naïve DB or conditioned starvation DB media, and plated under-buffer in microtitre dishes at varying cell densities. Here, cell aggregation was visually monitored after 24 h.

As seen previously [13–16], *Dictyostelium* have a very sharp cell-density threshold cut-off for aggregation/non-aggregation, regardless of media treatment used (Fig. 1; Additional file 1: Figure S1A,B). However, use of conditioned DB media for plated development of WT cells allowed aggregation at a 4-fold lower cell density than cells incubated in naïve media (Fig. 1; Additional file 1: Figure S1B). Additionally, we examined effect of conditioned media on cells lacking the signaling inhibitory, heterotrimeric G protein Gα9 (Additional file 1: Figure S1C [21]). *G*α*9*-null cells aggregate at a lower density than WT [21] and show greater sensitivity for aggregation with conditioned WT media, to a > 16-fold cell-density dilution effect (Additional file 1: Figure S1C). These data suggest that starved cells secrete and accumulate a large sized activity that alters cell-density sensitivity for development.

### Purification of a cell-density aggregation factor

**Fig. 2:**
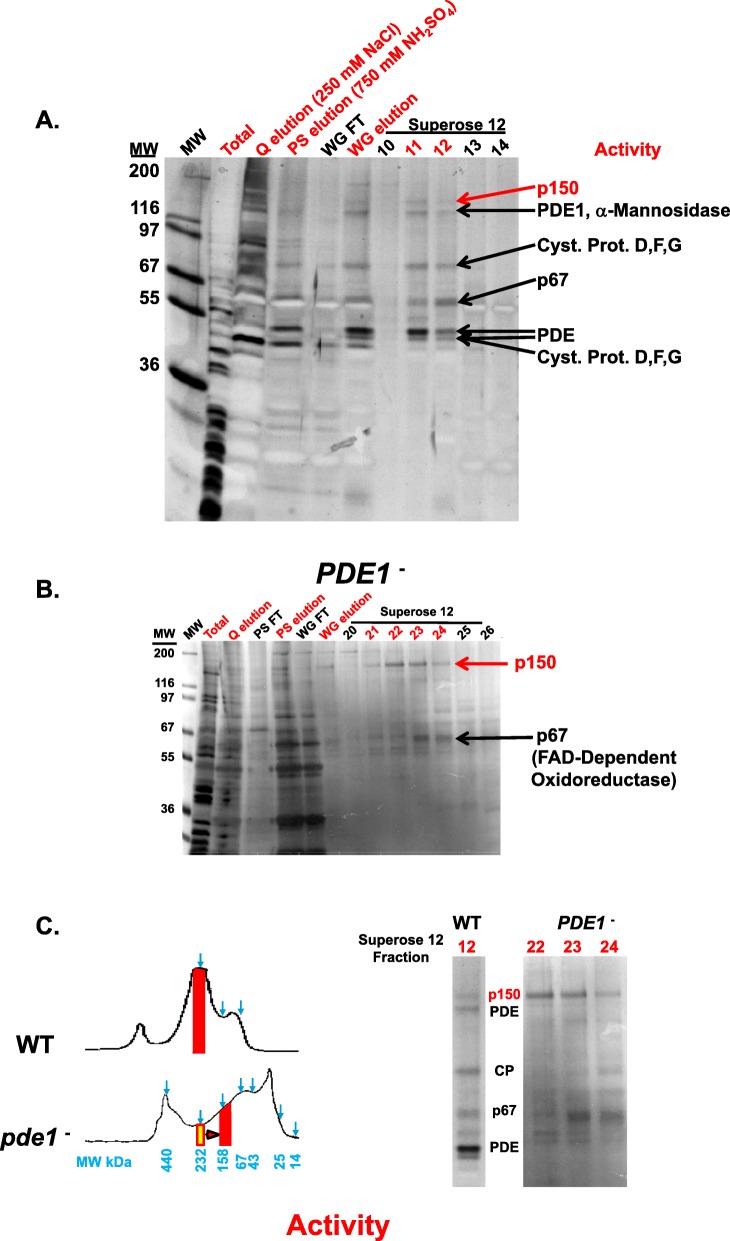
Purification of the density aggregation activity. **a** The cell-free, > 30-kDa conditioned media from WT cells were fractionated on mono Q, phenyl sepharose (PS), wheat germ agglutinin (WG), and Superose 12 columns. Fractions were assayed for density-dependent aggregation activity on WT cells at low cell density (< 20 × 10^3^ cells/cm^2^). Selected bound or flow through (FT) fractions separated by SDS gel electrophoresis are shown. Gels were stained with silver and protein bands indicated from Superose 12 fractions (e.g., 11 and 12) were used for peptide sequencing. Proteins matching each band are indicated (see Additional file 3: Figure S3). **b** The procedure followed Fig. 2a, but using conditioned DB from *PDE1*-null cells. Superose 12 fractions 22, 23, and 24 were used for MS/MS peptide sequencing. **c** Comparison of Superose 12 fractionations of conditioned DB from WT or *PDE1*-nulls cells, with protein profiles, relative MW positions, and activity position shift indicated. Relative fractionation differences for the proteins in Fig. 2a are also shown

The extreme sensitivity of *Dictyostelium* developmental aggregation to limiting cell density defined an ideal assay for purification of density sensing factors. The concentrated > 30-kDa conditioned DB media preparation was first subject to Mono Q anion exchange chromatography, and fractions were tested on WT cells at sub-aggregation densities (< 20 × 10^3^ cells/cm^2^). A density-dependent developmental aggregation activity bound strongly to Mono Q columns eluting at 250 mM sodium chloride (Fig. 2a; Additional file 2: Figure S2). The Mono Q eluted activity also bound strongly to phenyl sepharose (for hydrophobic interaction), fractionating in a step gradient at 750 mM ammonium sulfate (Fig. 2a; Additional file 2: Figure S2). Many secreted proteins in *Dictyostelium* are modified with glycosyl moieties [33], and the aggregation activity bound wheat germ agglutinin lectin and could be eluted with glucosamine. Fewer than 20 proteins were detected in this eluate by SDS gel electrophoresis (Fig. 2a). Finally, the activity was size-fractioned on Sepharose 12, where maximal activity was restricted to several fractions (Fig. 2a), at a MW of ~ 250 kDa.

When the proteins in the Sepharose 12 activity fractions were separated by SDS gel electrophoresis, 6 definitive protein bands were identified in sufficient quantities for MS/MS peptide sequence analyses. Each band (see Fig. 2a; Additional file 3: Figure S3) gave peptides that matched precisely with annotated proteins in *Dictyostelium* [34, 35]. Several well-characterized proteins were among these, including cysteine proteases, α-mannosidase, and PDE1 (PdsA), the secreted phosphodiesterase which degrades extracellular cAMP (Additional file 3: Figure S3A). Two additional novel proteins of 67 kDa (p67) and 150 kDa (p150) were identified. p67 is similar to FAD-dependent oxidoreductases (Additional file 3: Figure S3B); p150 has a small EGF-type domain, but is otherwise unique (Additional file 3: Figure S3C). Clearly, none of the proteins had molecular weights in the 250-kDa range, suggesting that several multi-protein complexes might be present in the Sepharose 12 fraction, with at least one with cell-density aggregation activity.

The cysteine proteases, α-mannosidase, and PDE1 do not have properties that are consistent with density sensitive aggregation. In fact, high doses of PDE1 decrease extracellular cAMP levels and so inhibit, rather than promote, low-density cell aggregation. We show directly that cells lacking PDE1 accumulate a density aggregation activity in our assay (Additional file 4: Figure S4), as do cells lacking secreted CMF (a developmental factor) and secreted CtnA (a Countin Factor complex protein), known secreted density factors [29, 36]. We suggest that a secreted density developmental aggregation activity is likely associated with either p150 or p67.

PDE1 is found in several multi-protein complexes in *Dictyostelium*, and often in non-specific associations [37]. We speculated that density-dependent aggregation activity from WT cells might fractionate in a large non-specific protein agglomerate involving PDE1 and, thus, fractionate differently in extracts from *PDE1*-null cells, which retains the cell density-dependent aggregation activity (Additional file 4: Figure S4). The most dramatic fractionation difference we observe using *PDE1*-null cell media is an elution shift of the density-dependent aggregation activity on Sepharose 12 from an apparent MW of ~ 250 kDa in WT media to a smaller MW fraction of ~ 150 kDa from *PDE1*-null cell media (Fig. 2b, c). When these fractions are examined by SDS gel electrophoresis, p150 and p67 are the most prominent proteins. MS/MS peptide sequencing confirmed identity to previous sequencing. The low relative abundance of p67 in activity fraction 22 suggests that p150 might more likely possess density-dependent aggregation activity. We term p150 as a developmental promoting factor (DPF) and sought to support this conclusion through loss-of-function and gain-of-function studies.

**Fig. 3:**
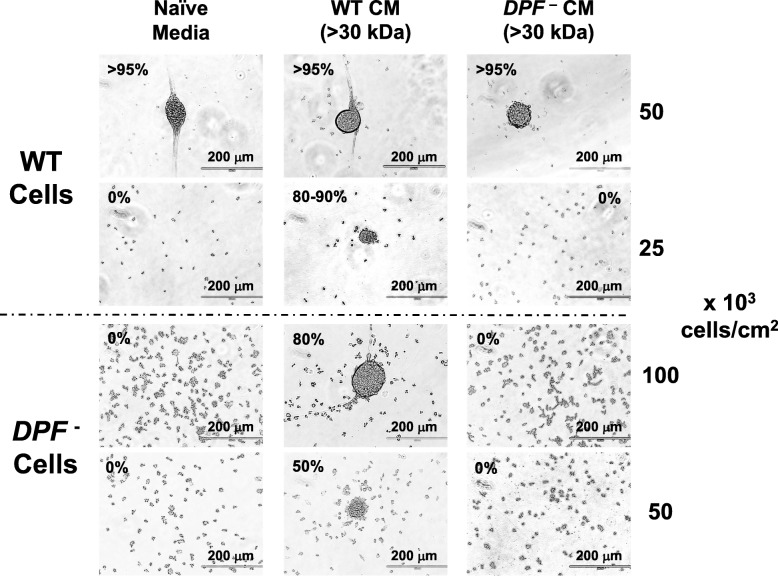
Cells deficient in p150 lack aggregation promoting activity. Log phase growing WT or *DPF*-null cells were adhered in a 12-well plate under DB starvation buffer at indicated cell densities for 24 h, using either fresh, naïve DB media or cell-free, > 30-kDa conditioned media from WT or *DPF*-null cells starved in DB for 18 h. Relative density-dependent activity for each is indicated as % aggregation. Scale bar = 200 μm

We studied p67 and p150 in loss-of-function studies by gene disruption (see the “Methods” section). Media from *p67*-null cells retain the density aggregation activity (see Additional file 4: Figure S4), suggesting that p67, a FAD-dependent oxidoreductase, is not involved in density-dependent development. However, conditioned media from cells with disruption in the p150 coding sequence (*DPF*^–^ cells) were unable to promote low density (e.g., 25 × 10^3^ cells/cm^2^) aggregation of WT cells (Fig. 3). Furthermore, the *DPF*-null, p150-deficient cells showed enhanced sensitivity to cell-density dilution for aggregation. Although aggregation of *DPF*-null, p150-deficient cells is inefficient at cell densities (e.g., 50 × 10^3^ cells/cm^2^) standard for WT cells, it can be restored using conditioned media from WT, but not from *DPF*-null cells (Fig. 3); still, the DPF-deficient cells seem less responsive to conditioned media than are WT cells (see below, Fig. 9d). Nonetheless, these data suggest that secreted p150 (i.e., DPF) is a previously uncharacterized protein that promotes early developmental events in *Dictyostelium*.

### DPF has secreted activity for promoting cell-density developmental aggregation

**Fig. 4:**
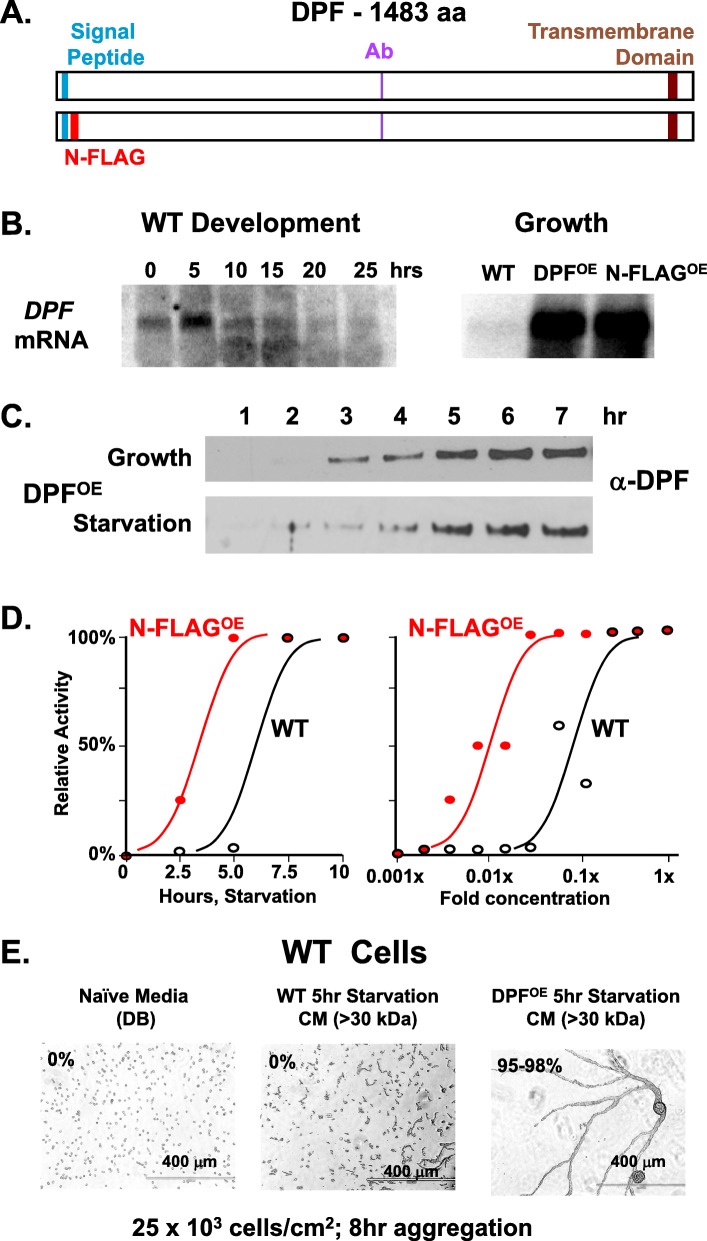
p150 protein structure, as termed DPF, and expression patterns. **a** Predicted structure of protein p150 (DPF). Full-length DPF has an N-terminal signal peptide and C-terminal transmembrane domain. An antibody was to a specific peptide (Additional file 3: Figure S3C). The relative positions these features are positioned along the 1483 amino acid backbone. The N-FLAG DPF expression construct was created with a FLAG peptide sequence inserted in-frame, 3′ to the signal peptide. **b** Left panel—developmental expression of *DPF* mRNA at indicated times for WT cells, using RNA-blot hybridization. Right panel—relative *DPF* mRNA levels in growing WT cells, WT cells expressing full-length DPF (DPF^OE^), or WT cells expressing full-length FLAG-tagged DPF (N-FLAG^OE^), using RNA-blot hybridization. **c** Log-phase growing WT cells overexpressing DPF (DPF^OE^) were transferred into fresh growth media or fresh DB starvation buffer at similar cell densities and supernatant fractions taken and immunoblotted to α-DPF (see Fig. 4a). **d** Left panel—growing WT or WT cells expressing full-length FLAG-tagged DPF (N-FLAG^OE^) were transferred into fresh DB. Cell-free media were taken at times indicated and tested for relative density-dependent aggregation activity, using WT cells at 20 × 10^3^ cells/cm^2^. Right panel—media collected at 7.5 h from both WT or N-FLAG^OE^ cells were diluted into fresh DB, as indicated, and tested for relative density-dependent aggregation activity, using WT cells at 20 × 10^3^ cells/cm^2^. Starting medium (1×) is 10-fold diluted, from a centricon concentrate of conditioned supernatant. **e** Log-phase growing WT cells were plated under DB buffer at 25 × 10^3^ cells/cm^2^ for 8 h, using either fresh, naïve DB media, or cell-free, > 30-kDa conditioned media from WT or DPF^OE^ cells starved in DB for 5 h (see Fig. 4d,e). Relative density-dependent activity is indicated as % aggregation. Scale = 400 μm

The annotated full-length protein for DPF is 1483 amino acids with a predicted N-terminal signal peptide and a C-terminal transmembrane domain (Fig. 4a; Additional file 3: S3C) that could anchor the protein in the plasma membrane. With processing of the signal peptide, a p160 fragment of DPF would be inserted into the plasma membrane as a single-pass protein. *DPF* mRNA is expressed during growth but is induced to high levels early in development (Fig. 4b), with maximal expression levels at 1–2 h after starvation on filters [38, 39]. Fusion of the WT DPF (DPF^OE^) or N-terminal FLAG-tagged DPF (N-FLAG^OE^) versions (see Fig. 4a) to an actin promoter overexpressed *DPF* mRNA > 10×, compared to WT cells (Fig. 4b). We also examined the time-dependent accumulation of secreted DPF after growing cells were transferred into fresh growth media or fresh DB starvation buffer at similar cell densities (Fig. 4c). The antibody used to detect DPF is not sufficiently sensitive to reproducibly quantify relative levels of WT protein (see below, Fig. 6c), but overexpressed DPF accumulates in extracellular media to ~ 10× greater level than for WT and at similar rates under growing or starved conditions.

Next, we showed that after shifting growing cells into DB starvation buffer, N-FLAG^OE^ cells (WT *Dictyostelium* cells, which overexpress N-FLAG DPF) accumulate a density-dependent aggregation activity several hours more quickly and to a higher level than do parental WT cells (Fig. 4d). These data suggest strongly that DPF is involved with a secreted cell-density developmental aggregation activity. Comparing cellular responses to media conditioned for only 5 h by WT or DPF^OE^ cells provides a finer level of experimental control. For some experiments below, DPF effects were more specifically defined by comparing responses to short-term (~ 5 h) accumulated conditioned media from WT or DPF^OE^ cells, which would, respectively, possess minimal or higher levels of DPF. Thus, 5 h accumulated media from DPF^OE^ cells will induce aggregation of low-density WT cells within 8 h, whereas comparable WT media is completely ineffective (Fig. 4e).

**Fig. 5:**
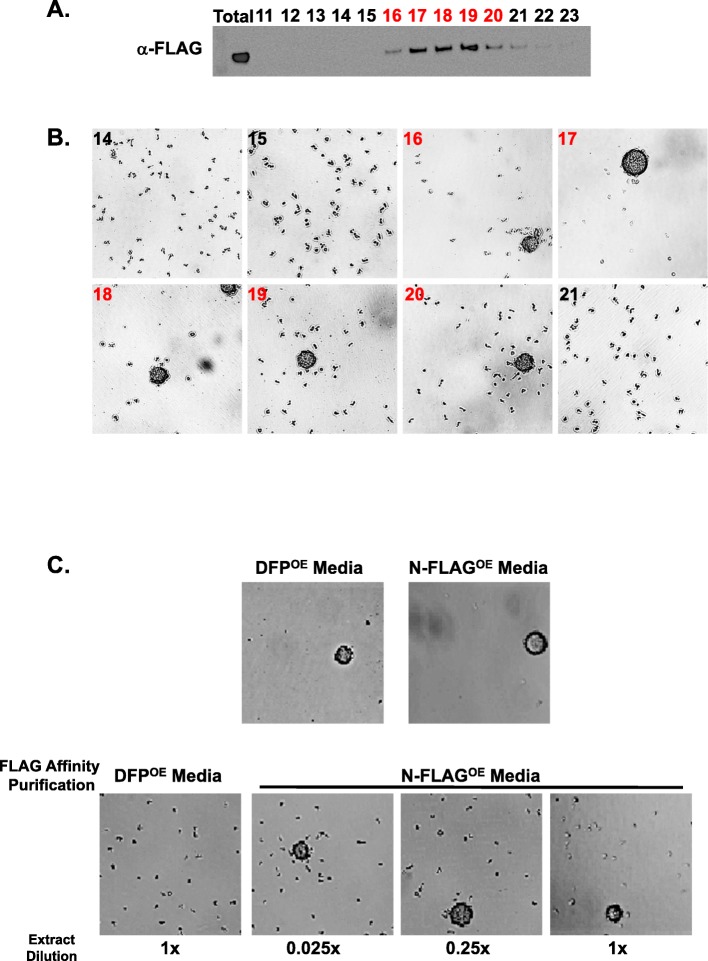
FLAG-p150 co-purifies with density aggregation activity. **a/b** Conditioned media from N-FLAG^OE^ cells was fractionated on mono Q and eluate fractions assayed by immunoblot for FLAG protein (**a**) and for density-dependent aggregation activity (**b**) using WT cells at 20 × 10^3^ cells/cm^2^. **c** Upper panels—Mono Q fractionated media from DPF^OE^ or N-FLAG^OE^ cells were assayed for density-dependent aggregation activity using WT cells at 20 × 10^3^ cells/cm^2^. Lower panels—media were affinity purified with α-FLAG and re-assayed at varying dilutions for density-dependent aggregation activity using WT cells at 20 × 10^3^ cells/cm^2^

Following, we fractionated media from N-FLAG-DPF overexpressing cells on Mono Q and tracked purification of the FLAG motif, by immunoblot assay, and in parallel, tested for density-dependent aggregation activity. We show that the N-FLAG-DPF protein eluted in MONO Q fractions 16–20 (Fig. 5a), in precise co-elution with density aggregation activity (Fig. 5b). We then prepared MONO Q fractions from WT cells overexpressing WT-DPF (DPF^OE^) and N-FLAG DPF (N-FLAG^OE^), and affinity-purified the fractions with α-FLAG-agarose (Fig. 5c). The fractionated media from both cell lines had similar starting density aggregation activity, but only the N-FLAG-variant of DPF showed enrichment to > 50× with α-FLAG-agarose (Fig. 5c). We conclude that the secreted DPF protein promotes developmental aggregation at limiting cell densities.

### Processed DPF is secreted by ectodomain shedding of a single-pass transmembrane protein

**Fig. 6:**
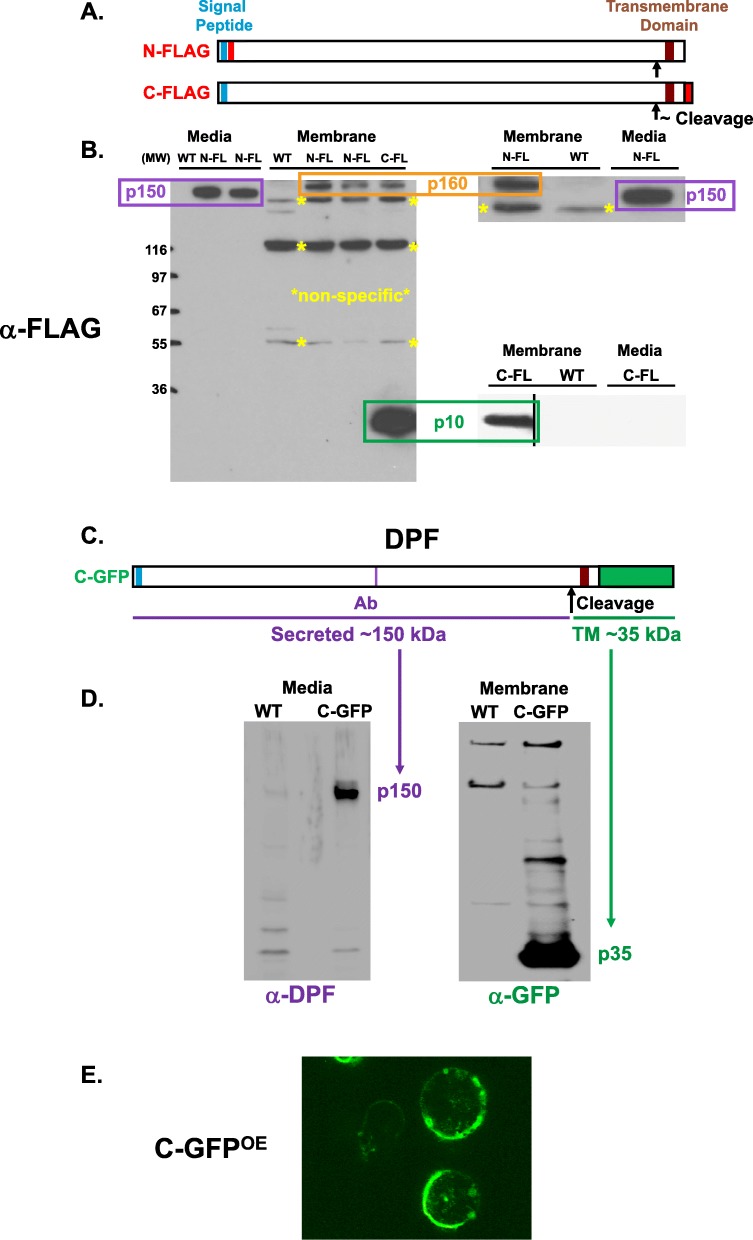
p150 is released from the plasma membrane by ectodomain shedding. **a** Two DPF constructs were engineered. One has an N-terminal FLAG (see Fig. 4a) and the other a C-terminal FLAG. **b** Cells expressing N-FLAG and C-FLAG were shaken in DB for 18 h and media and membrane fractions prepared and immunoblotted to α-FLAG. The most abundant N- and C-terminal tags are localized to separate sized fragments, suggesting processed cleavage for ectodomain shedding. A full-length DPF form is in the membrane as p160; it is processed to release p150 and membrane-anchored p10 (see Fig. 6a). **c** A C-terminal GFP DPF protein expression construct was also engineered. **d** Media and membrane fractions from WT cells and WT cells expressing C-GFP (C-GFP^OE^). Media proteins were immunoblotted to α-DPF (see Fig. 4a,c and Additional file 3: Figure S3C), and membrane proteins were immunoblotted to α-GFP. α-DPF detects secreted p150 and α-GFP detects membrane-anchored p10 fused to GFP. **e** Fluorescence localization of GFP in C-GFP expressing cells. Strong GFP fluorescence, as a read-out of the DPF TM domain, is seen at the cell periphery

The structure of DPF (see Fig. 4a) places it as a single-pass transmembrane protein with a long, glycosylated 150-kDa extracellular domain that is eventually secreted into the media. To understand the mechanism for secretion, we expressed N- and C-terminal FLAG-tagged variants of DPF and examined their cellular localizations (Fig. 6a). The N-terminal FLAG is seen associated with a 150-kDa protein in media preparations, but in a larger, 160 kDa, MW form in cellular membranes (Fig. 6b). The C-terminal FLAG is in two size variants in membranes, at 160 kDa and 10 kDa (Fig. 6a). The p10 variant is > 10× more abundant than the 160-kDa form. We suggest that the 160-kDa protein is a near, full-length transmembrane protein that has been signal peptide processed and glycosylated, and can be marked with both N- and C-terminal FLAG epitopes. Extracellular protease cleavage then releases a glycosylated p150 protein, which possesses DPF activity. Following ectodomain shedding, the residual 10-kDa protein remains membrane bound.

To examine processing differently, we expressed DPF with a C-terminal GFP tag (Fig. 6c). We see a strong p150 signal in media compared to WT cells using the α-DPF (Fig. 6d). A weak signal may correspond to the WT band (Fig. 6d). In membranes, we see C-terminal GFP fusion band at the expected MW of ~ 35 kDa. Most of the GFP staining is associated with the cell periphery (Fig. 6e).

### Gain-of-function activities in WT cells expressing high levels of DPF

**Fig. 7:**
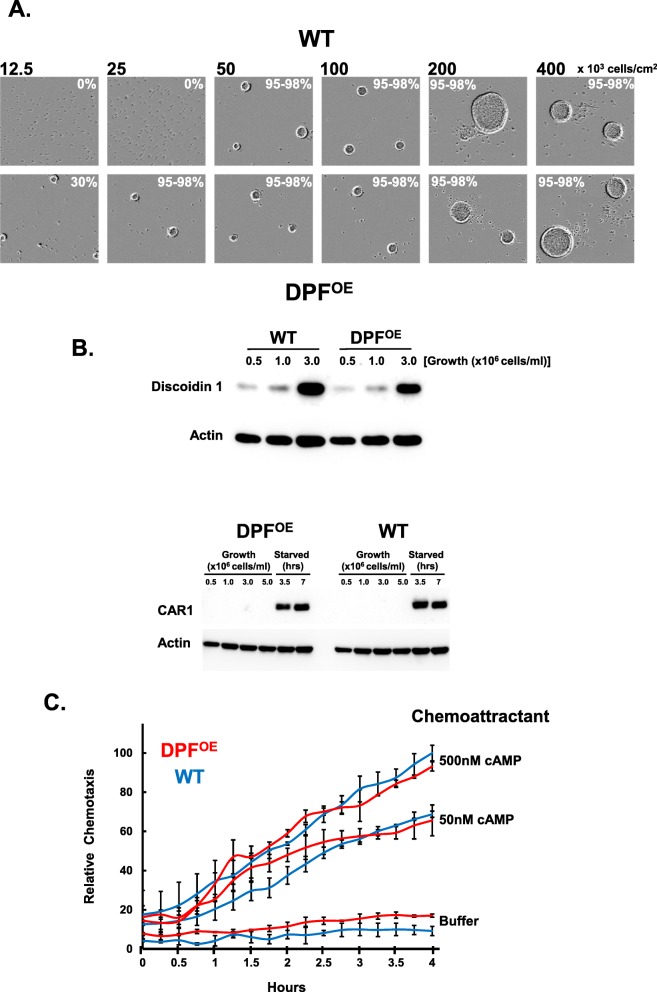
Gain-of-function studies of WT cells expressing high levels of DPF. **a** Log phase growing WT or DPF^OE^ cells were plated under-buffer at indicated cell densities for 24 h using fresh, naïve DB media. Relative aggregation efficiencies are indicated. **b** WT and DPF^OE^, *DPF*^-OE^ cells were grown to various cell densities in growth media and then identically starved as indicated. Cell lysates were prepared from cells at indicated times and immunoblotted to α-Discoidin 1, α-CAR1, and α-actin. **c** Time-course quantification of WT or DPF^OE^ cell migration to various doses of cAMP. Relative chemotaxis is normalized to WT cells at 500 nM cAMP at 4 h. Standard deviations are shown based upon three replicates

Next, we compared the behavior of WT cells and DPF^OE^ cells during aggregation. As expected for a density aggregation factor, DPF^OE^ cells are able to form aggregates at lower cell plating densities than for WT (Fig. 7a). Possibly, the DPF^OE^ cells become developmentally primed during growth in advance of WT and, thus, are able to initiate development at lower cell densities. To examine this, we looked for precocious expression of Discoidin 1 and CAR1 in DPF^OE^ cells, which are sensitive to pre-starvation induction as cell densities rise during growth [27, 28]. First, we show a large relative expression increase in Discoidin 1 protein levels with only a 3-fold increase in growth cell density. Nonetheless, expression levels of Discoidin 1 are largely similar comparing WT and DPF^OE^ cells (Fig. 7b). We see only limited expression of CAR1 at growing cell densities to 5 × 10^6^ cells/ml in both WT and DPF^OE^ cells, and no expression level differences between the two cell lines upon starvation (Fig. 7b). Thus, the data indicate that at starvation, DPF^OE^ and WT cells are at developmentally similar states.

We also studied chemotaxis of WT and DPF^OE^ cells to cAMP in the IncuCyte system [40, 41]. Cells were washed from media to remove exposure to endogenous DPF and then followed for chemotaxis for 4 h; during this time frame, significant levels of secreted DPF accumulate for DPF^OE^ cells, but not for WT cells (see Fig. 4d, e). Nonetheless, there were no statistically significant differences in migration during the course of the experiment between in WT and DPF^OE^ cells (Fig. 7c). Neither did we see a chemotaxis rate change for DPF^OE^ cells during the time course of the evaluation, indicating that accumulation of secreted DPF did not alter chemotactic sensitivity.

### Cells that overexpress DPF preferentially localize to aggregation centers and regulate prespore/spore patterns

**Fig. 8:**
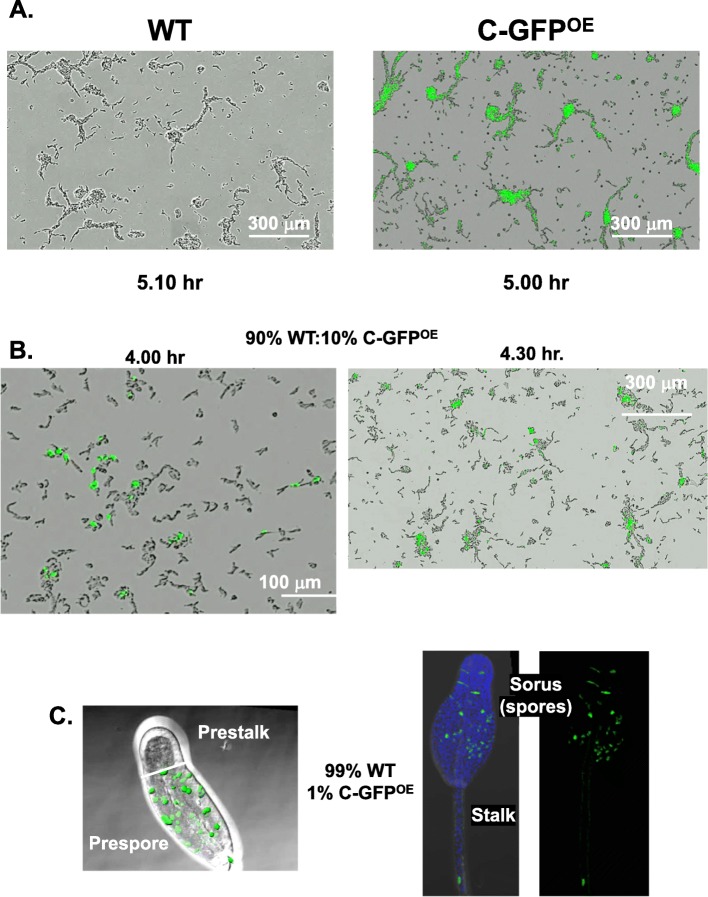
Cells that overexpress DPF regulate prespore/spore patterning. **a** WT or C-GFP^OE^ cells were identically plated on DB agar at a density of 400 × 10^3^ cells/cm^2^ for development and followed over time. Shown are similar time frame images including both DIC and GFP fluorescence. **b** A 9:1 mixed population of WT or C-GFP^OE^ cells were plated for development and followed over time. Shown are two time frame images including both DIC and GFP fluorescence (see Additional file 8: Movie S1). **c** A 99:1 mixed population of WT or C-GFP^OE^ cells were plated for development and developed to the slug stage (left) or to terminal differentiation (right). Shown are confocal images including both DIC and GFP fluorescence, with prespore/prestalk and spore/stalk regions indicated

As might be expected, WT and DPF^OE^ cells developed under high cell-density conditions (> 300 × 10^3^ cells/cm^2^) would both accumulate DPF at developmentally sufficient levels and, thus, exhibit similar patterns for developmental aggregation, in timing, territory area, and size of aggregates (Fig. 8a). However, when cells were developed in mixed cultures at a 9:1 ratio of WT to DPF^OE^, the DPF^OE^ cells preferentially localize at centers for aggregate formation (Fig. 8b; Additional file 8: Movie S1), suggesting that high levels of DPF facilitate cell-cell communication to enable developmental signal-response. Following aggregation, *Dictyostelium* cytodifferentiate into two major precursor cell types, prespore and prestalk cells, which become spatially segregated, along an anterior (prestalk) and posterior (prespore) axis [42] of the developing pseudoplasmodia. Previous work [21, 43] had shown that cells which establish signaling centers for aggregation are preferentially fated to prespore cytodifferentiation. We show that DPF^OE^ cells are not only locally enriched at centers for signaling, they also preferentially accumulate in prespore regions of the developing pseudoplasmodia and in spores during terminal differentiation (Fig. 8c), compared to WT and various controls (Additional file 5: Figure S5).

### Cell-autonomous functions for DPF

**Fig. 9:**
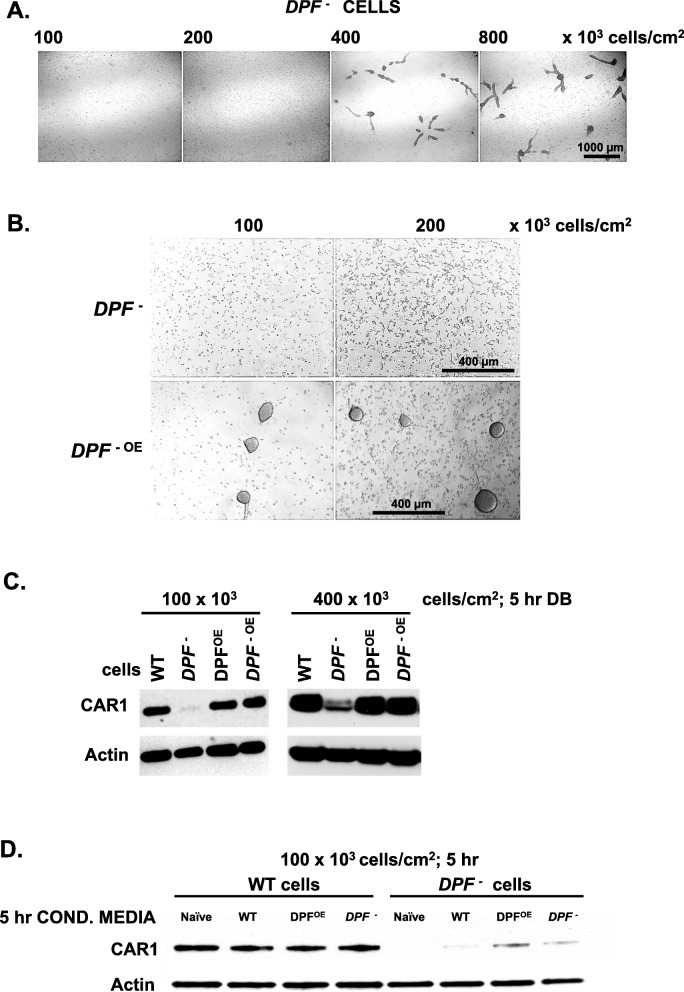
Cell-autonomous and non-autonomous functions of DPF in development. **a** Log phase growing *DPF*^-^ cells were adhered in a 12-well plate under fresh, naïve DB starvation buffer at indicated cell densities for 24 h. **b** Log phase growing *DPF*^-^ or *DPF*^-OE^ *Dictyostelium* were plated under fresh, naïve DB starvation buffer at indicated cell densities for 24 h. **c** WT, *DPF*^-^, DPF^OE^, and *DPF*^-OE^ cells were adhered in fresh, naïve DB media at a density of 100 × 10^3^ or 400 × 10^3^ cells/cm^2^ and developed for 5 h. Cell lysates were prepared and immunoblotted to α-CAR1 and α-actin. **d** WT and *DPF*^-^ cells were adhered at a density of 100 × 10^3^ cells/cm^2^ and developed for 5 h using fresh, naïve DB media, or > 30-kDa conditioned media from WT, DPF^OE^, or *DPF*^-^ cells following starvation in DB for 5 h. Cell lysates were prepared and immunoblotted to α-CAR1 and α-actin

Where WT cells are able to aggregate at densities of 50–100 × 10^3^ cells/cm^2^ (see Figs. 1, 3, and 7a; Additional file 1: Figure S1), cells deficient in *DPF* require densities > 200 × 10^3^ cells/cm^2^ for aggregation under buffer (Figs. 3 and 9a), supporting the role of DPF in density-dependent development. We also confirm a role for DPF in density sensing during standard developmental processes, where WT cells will aggregate at 4× lower cell density than will *DPF*-null cells on agar surfaces (Additional file 6: Figure S6A). Although WT and *DPF*-null cells both aggregate at 200 × 10^3^ cells/cm^2^ by 8 h (Additional file 6: Figure S6A), WT cells initiate the process several hours earlier (Additional file 6: Figure S6B); likewise, where WT and DPF^OE^ cells aggregate at 50 × 10^3^ cells/cm^2^ (Fig. 7a), DPF^OE^ cells initiate the process earlier (Additional file 6: Figure S6C). Nonetheless, at the high cell density, aggregated *DPF*-null cells can proceed through terminal stages of differentiation (Additional file 6: Figure S6D). Furthermore, re-expression of full-length DPF in *DPF*-null (*DPF*^-OE^) cells promotes aggregation at 3–4× lower cell densities (Fig. 9b). While conditioned media containing DPF is able to restore lower density aggregation of *DPF*-deficient cells (Fig. 3), it may not be as effective on *DPF*-null cells compared to WT. This may suggest that the TM/cytoplasmic domain of DPF may have a cell-autonomous function.

To better discern the role for membrane-bound DPF, apart from a secreted DPF form, we looked more clearly at development, using induced CAR1 expression as an essential marker read-out for early multi-cell development. CAR1 expression was monitored in *DPF*-null cells and DPF rescued *DPF*-null (*DPF*^-OE^) cells, under conditions restrictive (100 × 10^3^ cells/cm^2^) or permissive (400 × 10^3^ cells/cm^2^) to *DPF*-null aggregation. WT controls were monitored in parallel. For WT cells, CAR1 is similarly expressed irrespective of DPF expression levels at both cell-density conditions (Fig. 9c). However, results with *DPF*-nulls are quite distinct, although not unexpected. CAR1 is very poorly expressed under density conditions that are non-permissive for *DPF*-null aggregation (100 × 10^3^ cells/cm^2^); however, re-expression of DPF in *DPF*-null (*DPF*^-OE^) cells rescues CAR1 expression to WT levels (Fig. 9c). CAR1 expression is easily detected (the 2 resolved bands reflect known phosphorylation variants), under aggregation permissive conditions (400 × 10^3^ cells/cm^2^) for *DPF*-null cells, although at reduced levels relative to parental cells; full expression is restored in *DPF*-null by re-expression of DPF (i.e., *DPF*^-OE^ cells).

Next, we examined the effects of conditioned media with or without secreted DPF, on CAR1 expression at a density (100 × 10^3^ cells/cm^2^) permissive to WT cell aggregation but not to *DPF*-null cell aggregation. For aggregated WT cells, CAR1 expression is similar regardless of development in naïve DB, or in 5-h conditioned media from WT cells, *DPF*-null cells, or DPF^OE^ cells (Fig. 9d); here only media from DPF^OE^ cells contains significant levels of secreted DPF (see Fig. 4d, e). In contrast, CAR1 expression is relatively low in *DPF*-null cells developed in naïve DB, in 5-h conditioned media from *DPF*-null cells, or in 5-h conditioned media from WT cells with limited levels of DPF (see Fig. 4d, e). Significant CAR1 induction is observed with 5-h conditioned media from DPF^OE^ cells, but to levels below those of WT. By comparing data using full-length DPF overexpression or response to secreted DPF alone (Fig. 9d), we suggest that although cellular response to secreted DPF promotes aggregation and CAR1 expression, the TM/cytoplasmic domain of DPF, either as a full-length protein or as a truncated remnant following ectodomain shedding, may have cell-autonomous functions (see Fig. 11).

**Fig. 10:**
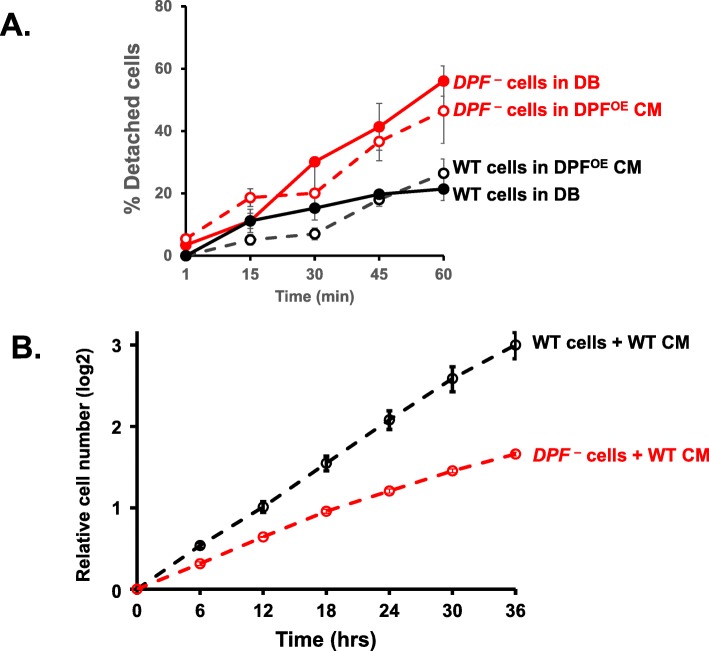
Cell-autonomous functions of DPF in adhesion and growth. **a** WT and *DPF*^-^ cells were adhered at a density of 400 × 10^3^ cells/cm^2^ to a six-well plate, washed and replenished with fresh, naïve DB media or 5-h conditioned media from DPF^OE^ cells. The dishes were then shaken at indicated time points at 90 rpm, and the percentage of de-attached cells quantified. Values indicate Mean ± SD from triplicate sets and three independent experiments. **b** Cell growth rates of WT and *DPF*^-^ cells in the presence of fresh growth media that was supplemented with DPF-containing conditioned growth media from WT cells. Growth rate was monitored at indicated time points. The values represent mean ± SD from three independent experiments

Indeed, we observe that *DPF*-null cells are more loosely attached to matrix surfaces than WT cells, perhaps partly explaining the different developmental effects of WT and *DPF*-null cells. We, therefore, quantified adherence to matrix surfaces using WT and *DPF*-null cells plated under naïve DB and under DB media conditioned for 5 h during starvation of DPF^OE^ cells. Plates with attached cells were shaken at a constant speed and detached cells quantified over time. *DPF*-null cells were clearly less adherent than WT, with no statistically significant effect of secreted DPF (Fig. 10a).

We also observed that *DPF*-null cells grew more slowly than did WT cells, with ~ 25% less volume on a collective cell basis (Additional file 7: Figure S7). Although growth rate and volume of *DPF*-nulls can be restored to WT properties by re-expression of full-length DPF (Additional file 7: Figure S7), *DPF*-null cells continue to grow more poorly than WT in fresh growth medium supplemented with conditioned growth media from WT cells that contain DPF (Fig. 10b). We conclude that DPF has a cell-autonomous function for adhesion and growth.

## Discussion

We have identified DPF, a protein that is secreted during the early stages of *Dictyostelium* development and functions to promote cell density-dependent development and consequently multi-cell aggregation. Cells at low density do not form aggregates under starvation-induced conditions; however, by increasing extracellular concentrations of DPF, through addition of purified DPF or by overexpression, cells will aggregate at > 4-fold lower densities than under standard conditions. In parallel, cells lacking DPF require higher cell density than do WT conditions for permissive developmental aggregation. We suggest that an extracellular threshold accumulation of DPF serves as an effective sensing factor to ensure that development proceeds when there are sufficient cell numbers for productive multi-cell formation and differentiation. Above this cell-density threshold, additional DPF would have limited influence. Furthermore, at even higher cell density, DPF is no longer essential for early development or terminal differentiation and fruiting body formation.

**Fig. 11:**
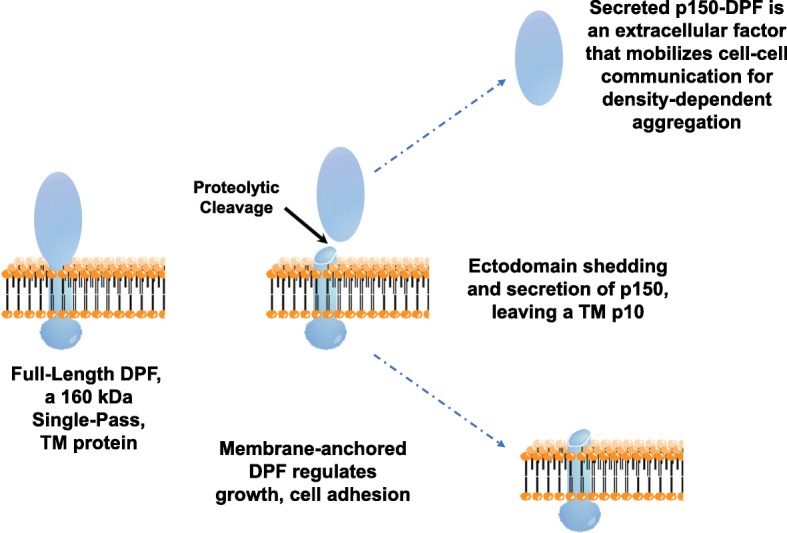
A model for ectodomain shedding and secretion of DPF. DPF is synthesized as an ~ 160-kDa protein that is inserted into the plasma membrane, following signal peptide cleavage. The transmembrane TM domain near the C-terminus anchors the single-pass DPF in the membrane. The long N-terminal ~ 150-kDa extracellular domain is glycosylated. Extracellular proteolytic cleavage, N-terminal to the TM domain, releases a p150 fragment into the media; The residual p10 TM/cytoplasmic fragment is retained in the plasma membrane. The secreted p150 possesses density-dependent aggregation activity and at high levels promotes aggregation at sub-optimal cellular densities and defines centers for aggregation. Membrane-anchored DPF has cell-autonomous activity for growth and adhesion

DPF is synthesized as a ~ 160-kDa protein, is inserted into the plasma membrane through an N-terminal signal peptide, and is then anchored by a C-terminal transmembrane domain as a single-pass protein (see Fig. 11). The long ~ 150-kDa extracellular domain is glycosylated and extracellularly released into the media by proteolytic cleavage and ectodomain shedding. Extracellular DPF then accumulates in proportion to relative cell density to mobilize cells for aggregation.

Unlike the Countin Factor (CF) Complex [30, 31], DPF does not appear to regulate group size. Where increasing concentrations of CF decreases the size of developmental aggregates, WT cells and cells overexpressing DPF form similarly sized aggregates at equivalent cell densities (see Fig. 8a). In addition, where the action of developmental factor CMF is dependent on starvation-induced secretion [36], DPF may be primarily regulated by transcriptional activation. DPF mRNA is rapidly induced at the onset of development [38, 39], and extracellular DPF accumulates at similar rates during both growth and development (see Fig. 4c).

The complexity of the developmental assay read-out (i.e., multi-cell aggregation) for DPF makes it difficult to understand potential DPF ligand interaction or a precise quantitative requirement. Since a 2-fold dilution of cells is sufficient to prevent aggregation, it is highly unlikely that biochemical responses to DPF occur in a strictly linear manner, but rather at a steep threshold sensing limit (see [2]). We also recognize that DPF cannot be the sole defining factor for cell-density aggregation. While other secreted factors certainly regulate aggregation sensitivity to cell density, our assay conditions more narrowly defined the influencing factors. In addition, regardless of DPF levels, cell-density dilution will reach a nadir below which cell-cell communication becomes ineffective, and distances for migration restrictive.

DPF studies with *g*α*9*-null cells [21, 44] may provide certain insight. Gα9 is an inhibitory Gα for cAMP receptor 1 (CAR1). Gα9 binds to CAR1, and cells deficient in Gα9 show increased synthesis of cAMP, faster developmental oscillations in cAMP, and low cell-density aggregation (see Additional file 1: Figure S1C) compared to WT cells [21]. Nonetheless, *g*α*9*-null cells are hyper-sensitive to DPF, aggregating with supplemented DPF at densities < 10-fold than that of WT (see Additional file 1: Figure S1C), indicating that DPF does not act through Gα9. It is interesting that both *g*α*9*-null cells and DPF^OE^ cells share another phenotype, suggesting perhaps that DPF may function in converging or parallel pathways. As an inhibitor of cAMP-signaling, *g*α*9*-null cells show more rapid cAMP-regulated development than WT and, accordingly, define centers that initiate cAMP-signaling when developed in mixed culture with a > 90% population of WT [21]; DPF^OE^ cells similarly show enriched signaling/aggregation center formation in mixed development with WT cells. Furthermore, cells that define aggregation centers are also fated for prespore cytodifferentiation during morphogenetic development [21, 43] and both *g*α*9*-null cells and DPF^OE^ cells show preferential prespore fate determination when developed in a predominant 99% WT population. However, our data do not indicate if DPF overexpression promotes the prespore/spore differentiation pathway or is inhibitory toward prestalk/stalk formation.

As in other systems, *Dictyostelium* utilize multiple extracellular receptor-signaling pathways for potential DPF targeting. GPCRs for several small molecule ligands (e.g., cAMP, folate, ATP) are known [40, 45–47], and secreted proteins CMF, CF, and chalones are suggested to function through distinct heterotrimeric G proteins [48–50]. The TgrB1/TgrC1 proteins represent a different class for cell-cell communication, as a ligand/receptor pair [51]. TgrB1 and TgrC1 are structurally related transmembrane proteins with long extracellular domains. These 2 extracellular domains exhibit physical interaction, with TgrC1 acting as an extracellular ligand that binds and activates the TgrB1 receptor [52]. Activated TgrB1 signaling is mediated through its C-terminal intracellular cytosolic domain [53]. We also recognize that DPF may facilitate cell-cell contact, in addition to or apart from ligand/receptor-dependent signal transduction, as a developmental priming event. Pathways may involve DPF-DPF homophilic recognitions, either as full-length/full-length DPF interactions and/or as secreted p150/full-length DPF interactions, or heterologous associations.

It is also not clear why centers for cAMP-signaling are enriched for DPF^OE^ cells. Secreted DPF may have diffusion limits, and if DPF functions in a path that promotes cAMP-signaling or cAMP persistence cells within an immediate area of highest DPF concentration may exhibit a more rapid and enhanced response for establishing these signaling centers, in contrast to more distal cells, and with possible reflection to morphogen gradients [7, 54, 55].

Regulation of transmembrane protein function by ectodomain shedding is complex and can release bioactive molecules that act both extra- and intracellularly [56]. Proteolytic cleavage (i.e., shedding) of single pass TM proteins, as for TNF and TGF (in true metazoa) and DPF in *Dictyostelium*, sheds an activated, extracellular ectodomain, but shedding can also create an alternative TM protein structure that is an available substrate for subsequent intra-membrane cleavage. Although in other systems released cytosolic domains may function as transcription factors, the cytoplasmic domain of DPF is very short (~ 30 amino acids; see Additional file 3: Figure S3C) and we do not see membrane release and cytosolic or nuclear accumulation of tagged-DPF C-termini. Possibly both the full-length and residual membrane-bound DPF fragments have distinct and specific roles, and certainly DPF exerts cell-autonomous effects that modulate cell adhesion, growth, and perhaps development.

## Conclusions

*Dictyostelium* grow in the wild as individual cells, but when they become starved for nutrients they are poised to enter a multi-cell developmental program. Multi-cell formation, however, is highly dependent upon cell sufficiency for productive developmental cell-cell communication and aggregation. We have identified the novel protein DPF in *Dictyostelium* that is secreted by ectodomain shedding and accumulates within the extracellular milieu in parallel with an increasing local cell population. In this manner, DPF serves as a density-sensing factor to correlate the developmental fate switch with the collective local cell population. Regions with the highest DPF concentration preferentially localize at centers for multi-cell formation and additionally determine cell-fate choice. We further demonstrate that DPF also has cell-autonomous functions, most probably associated with the TM/cytoplasmic region. Both segments of DPF, the secreted and the cell-inherent segments, regulate growth and developmental processes.

## Methods

### Cell lines and culture

*Dictyostelium* strains [34, 35] were confirmed and were grown axenically in D3T medium at 22 °C in suspension culture [57], at ~ 180 rpm, to a density of 1–1.5 × 10^6^ cells/ml; *DPF*-nulls were maintained and expanded as adhered cells on dishes. *ctnA*-, *CMF*-, *g*α*9*-, *p67*-, and *DPF*-null lines (see below) were grown under 10 μg/ml blasticidin selection. DPF overexpressing strains (DPF^OE^ and DPF^-OE^) and WT GFP cells were grown with 50 μg/ml G418.

### Preparation of conditioned media

To prepare conditioned media, log phase growing WT, DPF^-^, and DPF^OE^ cells were washed into DB and resuspended at 2 × 10^7^ cells/ml, at 22 °C with constant shaking (180 rpm) for 5–18 h. Cell supernatants were passed through the > 30-kDa cut-off filtration system [Centricon (MilliporeSigma)] to concentrate and remove smaller molecules. The concentrates were diluted to the original volume with DB buffer and used in aggregation and developmental assays.

To prepare growth conditioned media, cells were adhered at 10% confluency in a large petri dish, replenished with a fresh growth media and allowed to grow until ~ 70% confluency (~ 36 h). Media from growing culture was passed through the > 30-kDa cut-off filtration system, and the concentrates were diluted to the original volume with fresh growth media and used for growth rate studies.

### Density-dependent aggregation and development assay

For cell-density aggregation, log-phase growing cells were transferred to developmental buffer (DB) starvation media (10 mM phosphate, pH 6.4; 2 mM MgCl_2_; 0.2 mM CaCl_2_) and plated under buffer in microtitre dishes at varying densities, as indicated for each experiment. The plating buffer used was either untreated (naïve) DB, cell-conditioned DB (see below) from various cell lines, or varying media extracts at different purification steps, and used at undiluted or diluted strengths using untreated naïve DB.

For relative activity determination assays, cell density was below the threshold level for WT aggregation, generally ~ 20 × 10^3^ cells/cm^2^. Controls with naïve buffer were always performed in parallel, at both aggregation permissive (100 × 10^3^ cells/cm^2^) and non-permissive (20 × 10^3^ cells/cm^2^) densities. Relative aggregation was variable, and only semi-quantifiable; 3 separate experiments were performed, with similar result trends. In general, it is calculated as the sum area for cells in aggregates in a well plate compared to that at maximum aggregation, where fewer than 10% of input cells fail to aggregate.

To analyze development, growing cells in log phase (1–3 × 10^6^ cells/ml) were washed twice in DB buffer and developed on agarose at 400 × 10^3^ cells/cm^2^ (or as indicated), and images captured at time intervals [40, 58].

### Density aggregation factor purification

Cells were grown to log phase and transferred to DB buffer. 2000 ml cells were shaken for 18–24 h at 2 × 10^7^ cells/ml. Cell-free, > 30-kDa conditioned DB media were prepared by Centricon (MilliporeSigma) 30-kDa filtration, which concentrates to > 10×. Samples from starting preparations were reserved. Purifications were at 4 °C. Samples were adjusted to 1 mM PMSF and loaded onto a Mono Q column and eluted in a linear gradient from 10 mM to 1 M NaCl. Fractions were tested for activity, and the active fractions (~ 250 mM NaCl) were pooled and loaded to a phenyl sepharose column in 4 M NaCl in PB. Fractions were eluted in 250 mM NH_2_SO_4_ decreasing concentration steps from 1 M, and the active fractions (~ 750 mM NH_2_SO_4_) adjusted to 10 mM PB and loaded to wheat germ agglutinin. Bound protein was eluted in 0.5 M glucosamine, dialyzed into 10 mM PB, and loaded for Sepharose 12 fractionation.

Fractions were column adjusted to PB and tested for density aggregation activity, and the most active fractions used for additional analyses.

### SDS gel purification and MS/MS sequencing

The samples were resolved on a 3–8% gel, with prior reduction and alkylation, and visualized by silver staining (SilverQuest Silver Staining Kit, Invitrogen). Experimental and control gel bands were excised, de-stained, and washed according to SilverQuest. Proteins were subject to trypsin digestion, peptide extraction, and MS/MS peptide sequencing [59] as a contracted core component of Dr. Michael Kinter, Cleveland Clinic Foundation. Derived peptides were searched by BLAST within dictyBase [34, 35].

p67 peptide sequences match gene name *DDB_G0269892* in dictyBase [34, 35] and probably encodes an FAD-Dependent Oxidoreductase; p150 peptide sequences match gene name *DDB_G0289949* in dictyBase [34, 35] and encodes DPF, a developmental promoting factor.

### Gene constructs

Full-length coding *DPF* mRNA is ~ 4.5 kb. DPF was assembled in several parts by RT-PCR. Each fragment was sequenced, assembled into a single construct and also sequenced. The full-length cDNA was transferred into actin expression vectors [60]. The final assembled cDNA and fusion site for each construct was sequence confirmed.

p67 disruption was by homologous recombination of a blasticidin selection marker into an internal restriction fragment [61]. Gene disruption was determined first by PCR using multiple internal and external primers and confirmed by hybridization and sequencing. *DPF*-null cells (GWDI_26_C_3) were obtained and confirmed from GWDI (https://remi-seq.org) bank in dictyBase [34, 35]; disruption was within exon 2, at amino acid position 200, within the secreted extracellular domain.

### Immunoblotting

Whole cell lysates were prepared in Laemmli lysis buffer with 2.5% of β-mercaptoethanol and incubated at 95 °C for 10 min. Cell lysates were immunoblotted following gel electrophoresis (Bio-Rad, 4–20% tris glycine gels) with antibodies to DPF, FLAG (F3165, Sigma), GFP [57], CAR1 [62], Discoidin 1 (A. Kuspa, Baylor Medical College), and actin proteins. For CAR1 proteins, cell lysates were not heated. DPF antibody was mouse polyclonal, prepared (Genewiz) to a single peptide (see Additional file 3: Figure S3C).

### α-FLAG affinity purification

α-FLAG M2 affinity gel (F2426, Sigma) was used for binding, following the manufacturer’s instructions. Elution was for 1 h with FLAG peptide (F4799, Sigma) at 0.5 mg/ml and 1:1 bead volume ratio.

### RNA extraction and hybridization blotting

Total RNA was isolated using the Qiagen RNAeasy mini preparation kit [Qiagen # 74104] and following the manufacturer’s protocol. RNAs of equal quantity were separated on a 1.2% agarose–6% formaldehyde gels, blotted onto nylon membranes, and hybridized with cDNA probe labeled with [*α*-^32^P]dCTP [63].

### Chemotaxis

Growing WT and DPF^OE^ cells were transferred to 10 mM phosphate buffer, pH 6.5. Sixty microliters (~ 1 × 10^3^ cells) was added to the upper well of a chemotaxis plate (Essen Bioscience; cat # 4582200). Two hundred microliters of PB, with or without cAMP as indicated, was added to the bottom well, and the plate was transferred to the IncuCyte chamber, at 22 °C [35, 36]. Chemotaxis was recorded over time as a function of the cells imaged at the under surface of the membrane, using a × 10 objective lens; data were exported, analyzed, and graphed using Microsoft Excel [40, 41].

### GFP cell fluorescence

C-GFP DPF cells were shaken in DB and washed into phosphate buffer. Cells were plated on a glass bottom dishes (MatTek Corporation) and observed using the Axiovert 100 M (Carl Zeiss) inverted microscope [64].

### Membrane preparations

Cell suspensions at 8 × 10^7^ cells/ml in 10 mm Tris-HCl (pH 8), 1 mm MgSO_4_, 0.2 mm EGTA, and 10% glycerol were lysed by passage through a 5-μm nucleopore filter. Lysates were centrifuged at 4 °C for 30 min [65].

### Cell-matrix surface adhesion assay

Cell adhesion assays were performed as described by [64] with slight modification. Log-phase growing cells were adhered at a density of 400 × 10^3^ cells/cm^2^ in a six-well plate, washed, and replenished with DB or conditioned DB media. The dishes were then shaken at indicated time points at 90 rpm, and the percent of de-attached cells quantified [Cellometer Vision-Nexcelom Bioscience].

### Cell growth rates assay

To measure the cell growth rates, we adhered 5 × 10^3^ cells/well in a 12-well plate, replenished with fresh growth media with or without DPF and incubated at 22 °C. Growth kinetics were monitored in an Incucyte chamber, imaged using × 10 objective lens; data were exported, analyzed, and graphed using Microsoft Excel.

### Cell volume quantification

We measured packed cell volume as a relative estimate of comparative cell size. WT, DPF^-^, DPF^OE^, and DPF^-OE^ cells were grown in a dish culture until 70% confluency. Cells were collected in fresh media and diluted to a density of 5 × 10^6^ cells/ml. One milliliter of cell suspension was transferred to the Packed Cell Volume tubes (catalog # TPP-87005), spun at 3000 RPM for 1 min. The graduated scale on PCV tubes estimates the relative changes in cell volume.

## Supplementary information

**Additional file 1: Figure S1.** Conditioned media promotes aggregation at low cell density. A. Log-phase growing WT *Dictyostelium* were plated under DB starvation buffer at indicated cell densities for 24 hrs, using fresh, naïve DB media. Each immaged mass at 50 x 10^3^ cells/cm^2^ represents an individual cell aggregate grouping, not an individual cell. No aggregates are seen at 25 x 10^3^ cells/cm^2^. Distinction between single and aggregated cells is more easily seen in 3.5x-enlarged figures below, with aggregation efficiencies indicated. B. Log-phase growing WT *Dictyostelium* were plated under DB starvation buffer at indicated cell densities for 24 hrs, using either fresh, naïve DB media or cell-free, >30 kDa conditioned media from WT cells starved in DB for 18 hrs, with aggregation efficiencies indicated. Scale bar = 200 μm. C. Log-phase *g*α*9*-null cells were plated under DB starvation buffer at indicated cell densities for 24 hrs, using either fresh, naïve DB media or cell-free, >30 kDa conditioned media from WT cells starved in DB for 18 h.

**Additional file 2: Figure S2.** Column purifications for density aggregation activity. Mono Q and phenyl sepharose fractionations of conditioned media from WT for density-dependent aggregation activity (see Fig. 2a). Protein profiles are indicated, with relative salt concentration elutions.

**Additional file 3: Figure S3.** Peptide sequence matching. Peptide sequences from Fig. 2a,b,c were compared to all *Dictyostelium* proteins. Shown are deduced proteins for (A) PDE1, (B) p67 (an FAD-dependent oxidoreductase), and (C) DPF (Development Promoting Factor). Amino acid color symbols are indicated.

**Additional file 4: Figure S4.** Log-phase growing WT cells were plated under DB starvation buffer at 20 × 10^3^ cells/cm^2^ with fresh, naïve DB media or cell-free, >30 kDa conditioned media from the indicated cell lines starved in DB for 18 h.

**Additional file 5: Figure S5.** Left: A 1:99 mixed population of WT GFP or DPF^OE^ cells plated for development to the slug stage. Right: A 100% population of C-GFP^OE^ cells plated for development to terminal differentiation. Shown are confocal images including both DIC and GFP fluorescence, with prespore/prestalk and spore/stalk regions indicated.

**Additional file 6: Figure S6.** DPF is required for density-dependent aggregation but not terminal differentiation. A. Log-phase growing WT and *DPF*-nulls cells were placed on DB starvation buffer agar plates at varying cell densities and aggregation visually monitored at 8 hr. B. Log-phase growing WT and *DPF*-nulls cells were placed on DB starvation buffer agar plates at 200 x 10^3^ cells/cm^2^ and aggregation visually monitored at 5 hr. C. Log-phase growing WT and DPF^OE^ cells were placed on DB starvation buffer agar plates at 50 x 10^3^ cells/cm^2^ and aggregation visually monitored at 5 hr. D. WT and *DPF*-null cells were developed on DB agar for 24 hr. Images at comparable magnification show terminal fruiting body formation, with similar stalk/sorus size ratios.

**Additional file 7: Figure S7.** Growth rates and cell volume of WT and *DPF*^-^ cells. A. Cell growth rates of WT, DPF^OE^, and *DPF*^-^ and DPF^-OE^ cells monitored at indicated time points. The values represent mean ± SD from three independent experiments. B. The relative packed cell volume of log-phase growing WT, *DPF*^-^, DPF^OE^, *DPF*^- OE^ cells in growth media. The relative cell volume was measured using Packed Cell Volume (PCV) tubes and presented as percentage relative to WT cells. Data from three independent experiments are shown for each.

**Additional file 8:** **Movie S1.** A 9:1 mixed population of WT and C-GFP^OE^ cells were plated for development and followed over time (see Fig. 8b).

## Data Availability

Cell lines and vectors are available or accessed at dictyBase (http://dictybase.org). Most notably, DPF is *DDB_G0289949* [34, 35], and the *DPF*-null cell line is mutant GWDI_26_C_3 [34, 35]; GWDI mutants can be requested through the stock center (http://dictybase.org/StockCenter/StockCenter.html) within dictyBase.
