# Supplementary figures and images for: DPF is a cell-density sensing factor, with cell-autonomous and non-autonomous functions during Dictyostelium growth and development
Source: BMC Biol. 2019 Dec 2;17:97. doi: 10.1186/s12915-019-0714-9 (PMC6889452; doi:10.1186/s12915-019-0714-9)

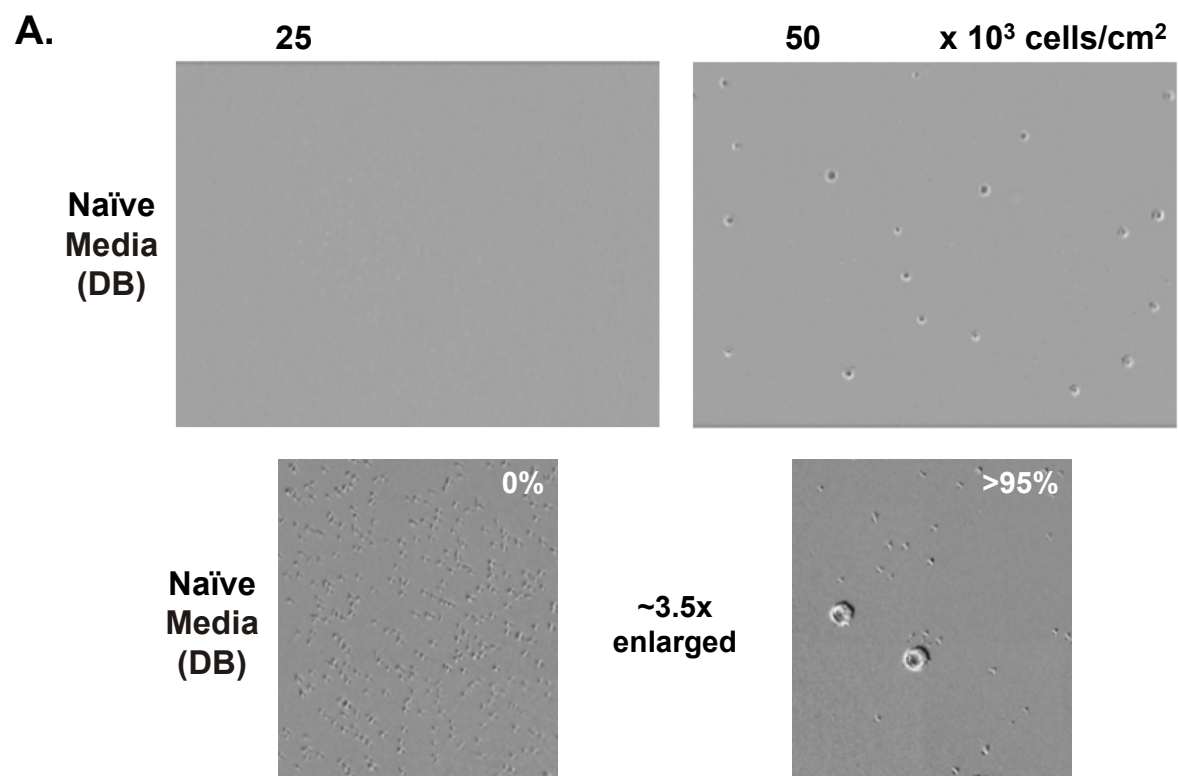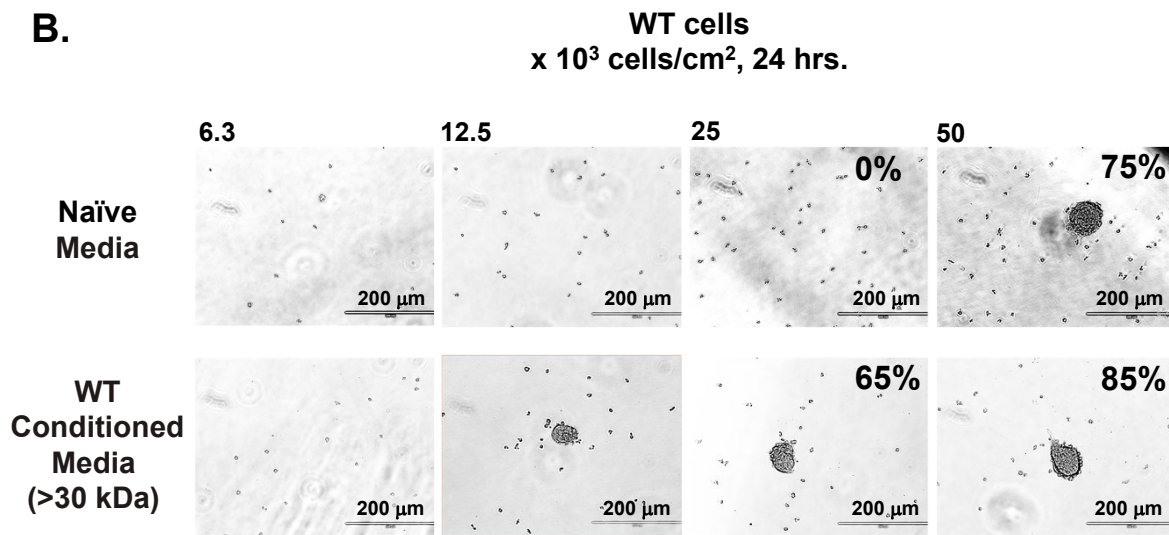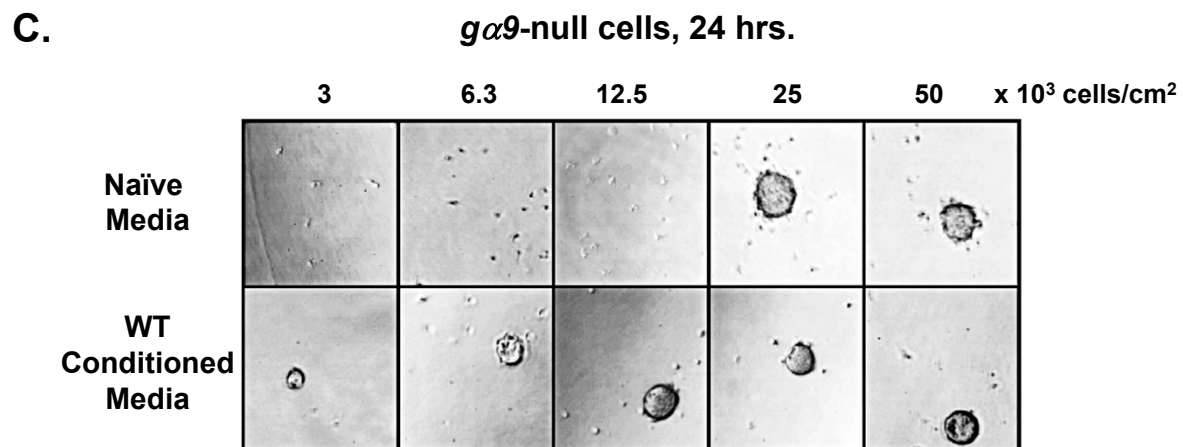

**Figure S1**

Supplement: Supplementary file 1 — Additional file 1: Figure S1. Conditioned media promotes aggregation at low cell density. A. Log-phase growing WT Dictyostelium were plated under DB starvation buffer at indicated cell densities for 24 hrs, using fresh, naïve DB media. Each immaged mass at 50 x 103 cells/cm2 represents an individual cell aggregate grouping, not an individual cell. No aggregates are seen at 25 x 103 cells/cm2. Distinction between single and aggregated cells is more easily seen in 3.5x-enlarged figures below, with aggregation efficiencies indicated. B. Log-phase growing WT Dictyostelium were plated under DB starvation buffer at indicated cell densities for 24 hrs, using either fresh, naïve DB media or cell-free, >30 kDa conditioned media from WT cells starved in DB for 18 hrs, with aggregation efficiencies indicated. Scale bar = 200 μm. C. Log-phase gα9-null cells were plated under DB starvation buffer at indicated cell densities for 24 hrs, using either fresh, naïve DB media or cell-free, >30 kDa conditioned media from WT cells starved in DB for 18 h. [file 12915_2019_714_MOESM1_ESM.pdf]

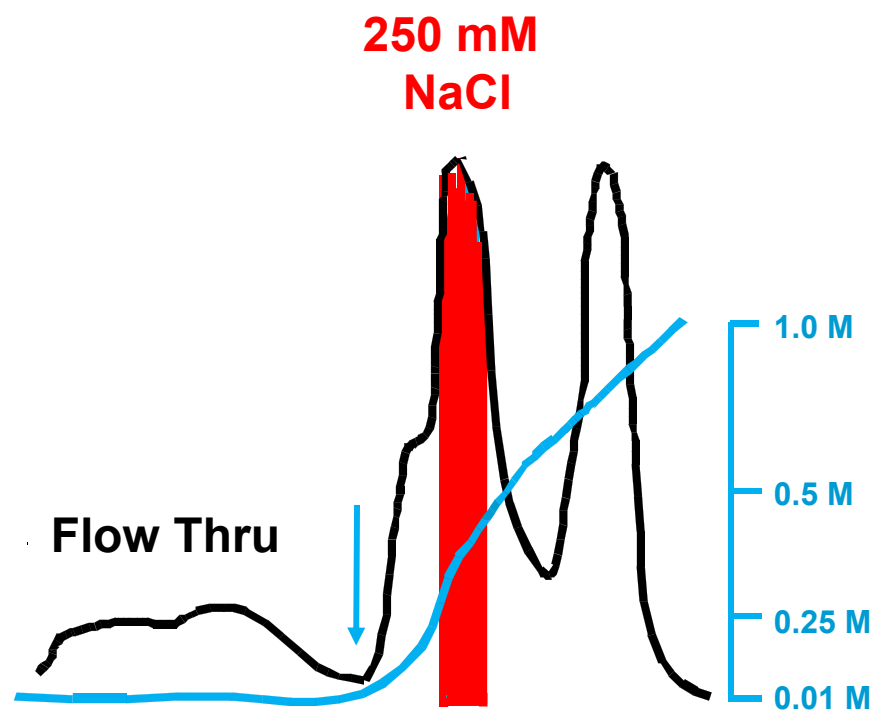

**Mono Q**

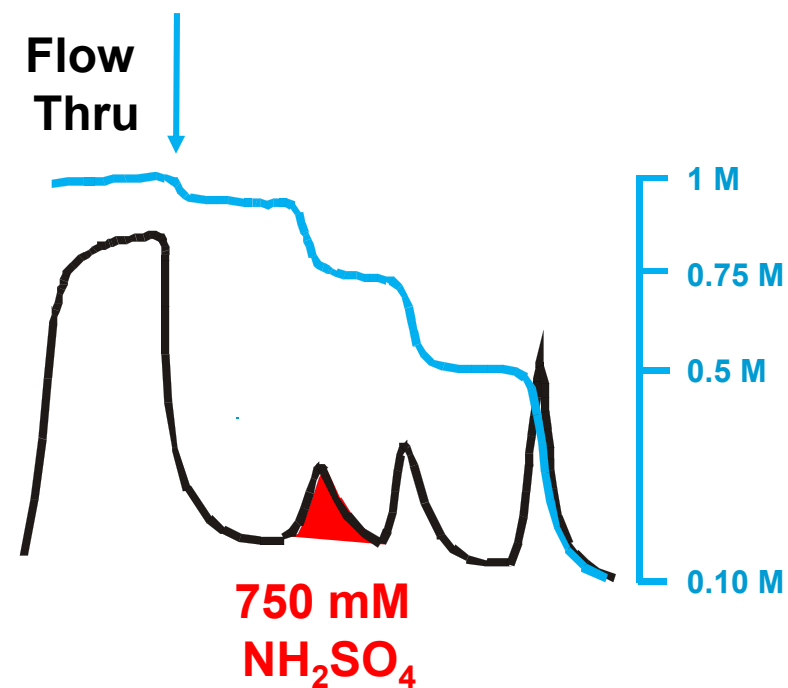

**Phenyl Sepharose**

**Activity Peaks**

**Figure S2**

Supplement: Supplementary file 2 — Additional file 2: Figure S2. Column purifications for density aggregation activity. Mono Q and phenyl sepharose fractionations of conditioned media from WT for density-dependent aggregation activity (see Fig. 2a). Protein profiles are indicated, with relative salt concentration elutions. [file 12915_2019_714_MOESM2_ESM.pdf]

## WT Cell Aggregation

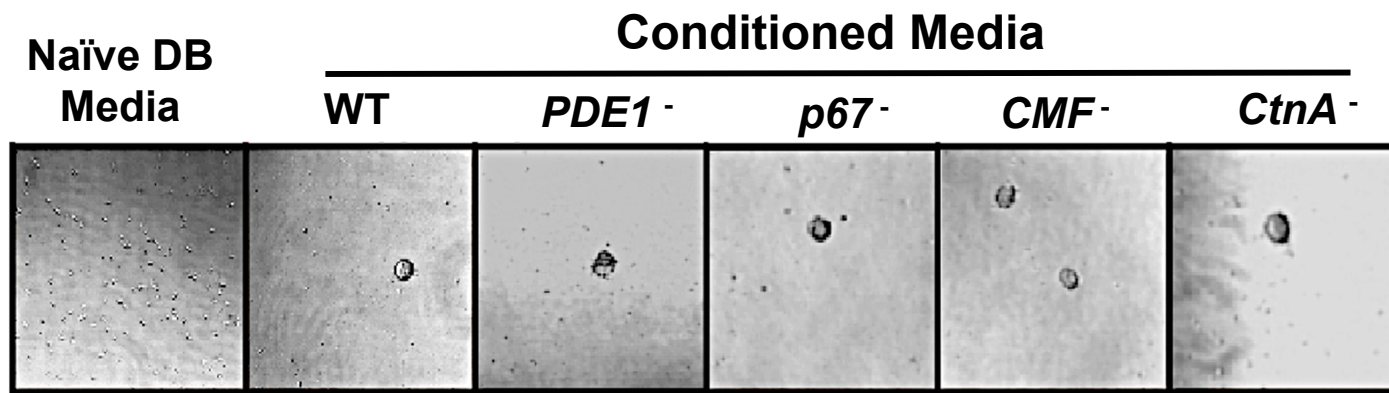

Figure S4

Supplement: Supplementary file 4 — Additional file 4: Figure S4. Log-phase growing WT cells were plated under DB starvation buffer at 20 × 103 cells/cm2 with fresh, naïve DB media or cell-free, >30 kDa conditioned media from the indicated cell lines starved in DB for 18 h. [file 12915_2019_714_MOESM4_ESM.pdf]

**1% WT GFP:99% DPF<sup>OE</sup>**

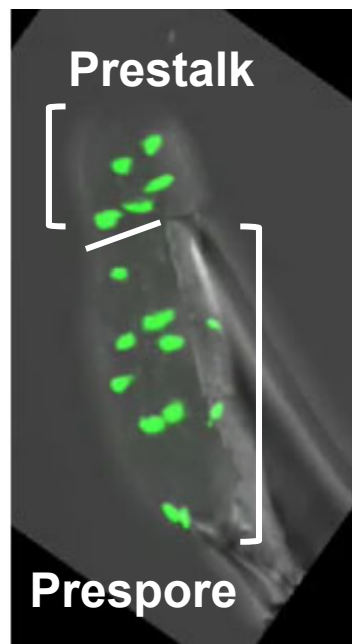

**100% C-GFP<sup>OE</sup>**

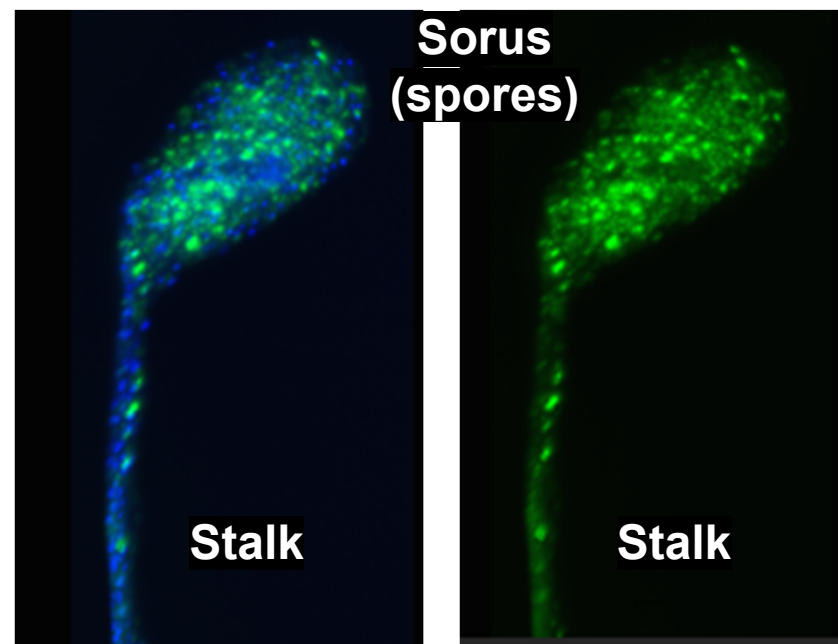

**Figure S5**

Supplement: Supplementary file 5 — Additional file 5: Figure S5. Left: A 1:99 mixed population of WT GFP or DPFOE cells plated for development to the slug stage. Right: A 100% population of C-GFPOE cells plated for development to terminal differentiation. Shown are confocal images including both DIC and GFP fluorescence, with prespore/prestalk and spore/stalk regions indicated. [file 12915_2019_714_MOESM5_ESM.pdf]

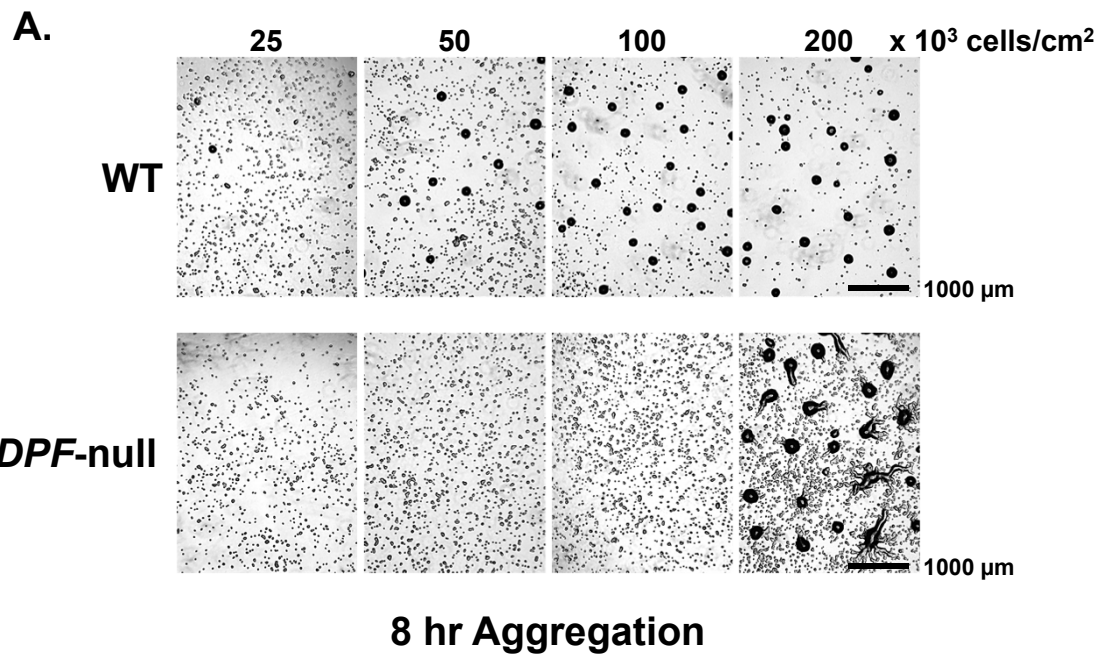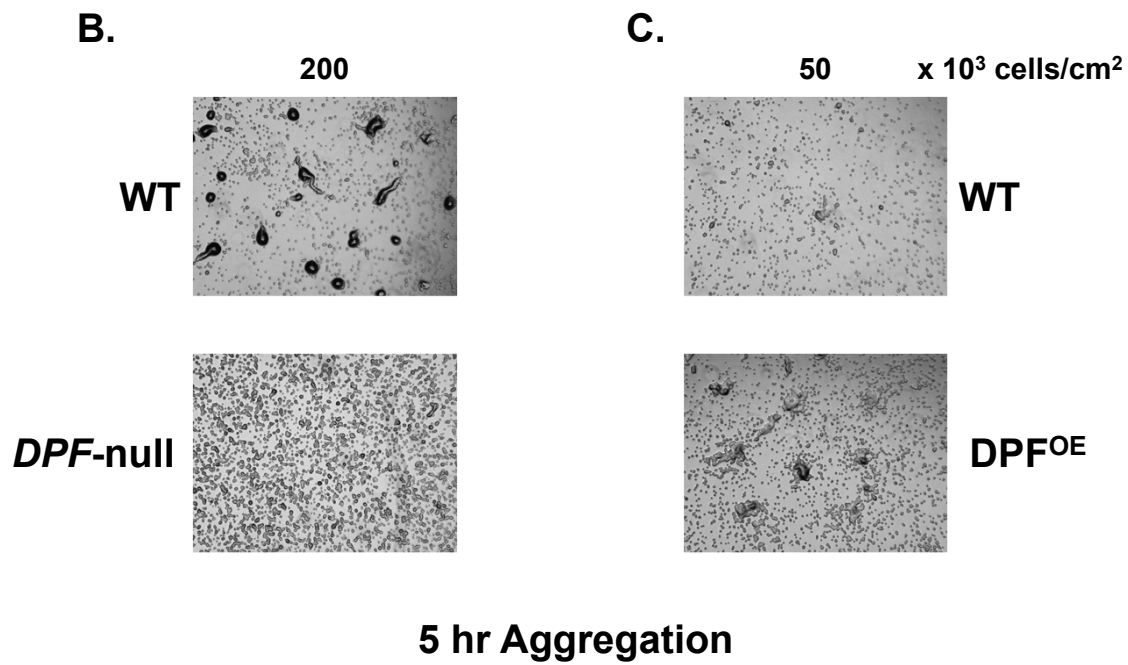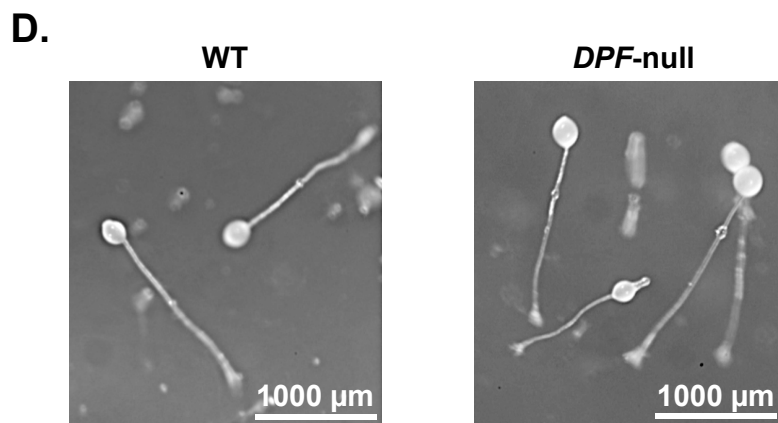

**Figure S6**

Supplement: Supplementary file 6 — Additional file 6: Figure S6. DPF is required for density-dependent aggregation but not terminal differentiation. A. Log-phase growing WT and DPF-nulls cells were placed on DB starvation buffer agar plates at varying cell densities and aggregation visually monitored at 8 hr. B. Log-phase growing WT and DPF-nulls cells were placed on DB starvation buffer agar plates at 200 x 103 cells/cm2 and aggregation visually monitored at 5 hr. C. Log-phase growing WT and DPFOE cells were placed on DB starvation buffer agar plates at 50 x 103 cells/cm2 and aggregation visually monitored at 5 hr. D. WT and DPF-null cells were developed on DB agar for 24 hr. Images at comparable magnification show terminal fruiting body formation, with similar stalk/sorus size ratios. [file 12915_2019_714_MOESM6_ESM.pdf]

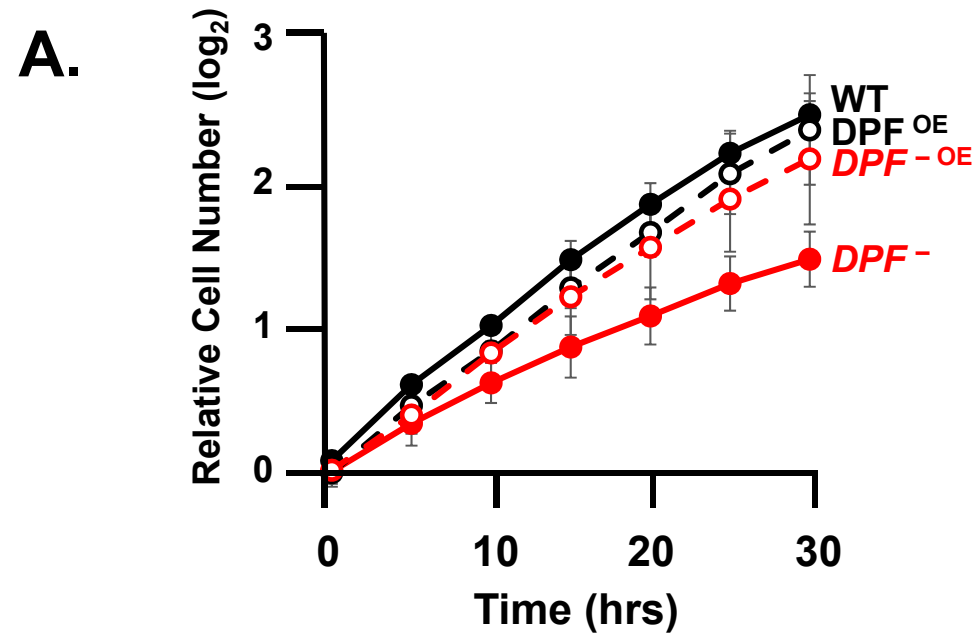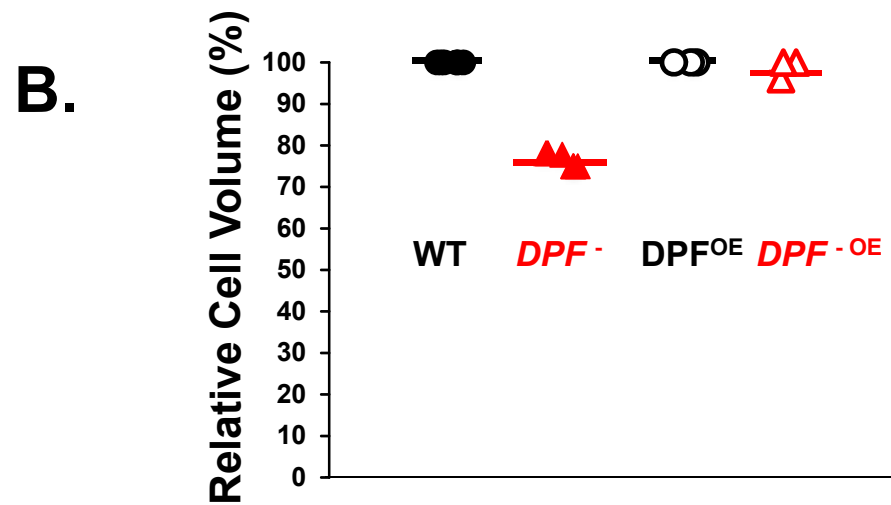

Figure S7

Supplement: Supplementary file 7 — Additional file 7: Figure S7. Growth rates and cell volume of WT and DPF- cells. A. Cell growth rates of WT, DPFOE, and DPF- and DPF-OE cells monitored at indicated time points. The values represent mean ± SD from three independent experiments. B. The relative packed cell volume of log-phase growing WT, DPF-, DPFOE, DPF- OE cells in growth media. The relative cell volume was measured using Packed Cell Volume (PCV) tubes and presented as percentage relative to WT cells. Data from three independent experiments are shown for each. [file 12915_2019_714_MOESM7_ESM.pdf]
