# Supplementary material for: DPF is a cell-density sensing factor, with cell-autonomous and non-autonomous functions during Dictyostelium growth and development
Source: BMC Biol. 2019 Dec 2;17:97. doi: 10.1186/s12915-019-0714-9 (PMC6889452; doi:10.1186/s12915-019-0714-9)
Supplement: Supplementary file 3 — Additional file 3: Figure S3. Peptide sequence matching. Peptide sequences from Fig. 2a,b,c were compared to all Dictyostelium proteins. Shown are deduced proteins for (A) PDE1, (B) p67 (an FAD-dependent oxidoreductase), and (C) DPF (Development Promoting Factor). Amino acid color symbols are indicated. [file 12915_2019_714_MOESM3_ESM.pdf]

A.

PDE1/PdsA

MALNKKLISLLLLIFIILNIVNSHQQEDCDDDDEDIGISAERSERRSVKNSNDGSNF
YNLNDYYTPENWNYYSGSFATKDCRDASYITIPLGTTGGLDEGNLSSFLLTKK**GSNL**
FIALDAGTVWQGVRRLTTFKYFNTLFNITYPSWAVLPEQRTSWFLKNHVMSYFIGHS
HLDHVGGLILVSPEDYLAKNWIDVQPPINNGIMGLIRK**LGFKPTDFTSSSILQK**KTI
MGLPSTINSISTNLFNNQVWPNLPSFGRYQYFSLASGIEYPFTELVPYNATTMSLVA
NEFPFSVKVKPFELCHDNLISTSFLFTDSISGEQIAFFSDTGVPSSVACDWEGKIYA
VWKQIKIDKLKAIYIETSFPNNTPDSAMFGHLRPRDVMK**LMDQLLVQSIQTSPPMTN**
LKHVK**LIIEHIK**P**QVAEDPNGWTTQR**VIYQQLKEANNNGVRIIIPNQGDPICI0

B.

p67
(FAD-Dependent Oxidoreductase)

MNKILILFICVLSCFINSAQSLTLPQLTAQINGKVI**SQSSPDFNNARFGYNYR**YNRVPQI
IVQPLDTASVVLALEYAQTNNLLVSVKSGGH**SAIAEGVQDLR**VV**IDVSQMK**QI**SYDPVSN**
IITTQSGNKWVEVYNYTINQHQVATPGGSCPSVSVGGLTLGGGANDLSTVHGLATDNVVE
LEVVLANRSVVIANEQTNVDLFWALRGGGHGGFGIVTLFKFRAHPVLPTYYSAWITYAWS
DFEDVLTYVNTFSETMPNTVNLYFTAWRSSNATNPSVALSCFFNGPSQLGESHCGSFRNF
SGSKRNTYITPVTISVTNASYYETVKNGTDPKAR**SSYTSGSFLTGIDK**KVIKNIKTQLEK
SP**VTPYTFNTAR**LNLYWQGGQMLNQAR**DYNAYVHR**TYPWNAVWLVSYTGGEFEEPYRKWI
KQTYRKFESFSEGVYQNYPDENNLDNWAEAYYMENYPKLQVVKAT**YDPNNYFR**FAQSIGS
PSLSS

C.

Full-Length DPF
(Development Promoting Factor)

MKQ**IIFLFFSLLLFNCFLKTNGD**FQVLNQTTVSTTSMYYNMGSVIGCYTTILIEIPFNSTFGQIKK**FTVF**
ASESNTFRIQ**SAIPLNQDTYK**IDGTILNLGSYSISLNGSYANNATEKITPIDLTCSDAPNITDLGIVLPS
ESIPISRYGEYLFEVPVIVPALTESDSIELSFALNESSPFKIFYEQISTNFFILRFKLNDPSLISFQGPL
EITPQNLNSGVIIKSYTSFIVYPNVGVSSESQFSSIYFYPDYNGETTSEYFGYVTFDVLNDDRAI**VPGVT**
NNLLNLDTSEKMDFGVAFPVGYNGTHSQFMGIIEPGRKASFYWYLASTDNSDILHTRFVPKRDVRPVPIS
TSAQELNYCRIGIIVVGDQSFDYTMPTIYKFSTNNGDFHEYIPYWSVDSFYSNTSITSISHELILPAGYY
NPTFKMYASILNYDDTVSLQVPGSPVPPLGNRAPPTVLDIELISPTGILSNFYILRFKVQSDYSEFHHLD
INGKKVYAINIVSGSLSQGSIGYYEINVNYTFIKSLSNSFTISACGIFLDCQTNQYFPQLLNPFPSLQLP
HNNLITVYNITYINFKYNNLNTTNTSR**NNIIYLK**INGLSGSGDAET**IQFK**LDIYDDYLPFTWNSLLSMFQ
CNFKIPGNYYSGNVGYSIILGSNSFSYSSVFIATGSNSTLNVTSLKSDMLPPIINYIQAYPTTNIYLDPY
NSSLNIFGWDLEIEEGVNGFDHGEVNITSAYDKVGFTFKFNSSDLIDGYIIEIRINHTEMVCLDQAYSIT
SVYL**VDKQGYETSRSSQY****QSVSALFK**VAASDQLLIYVQCSNDNGIGYNSTHPDVITPFIYSFFLDSGLFD
IQLDYGSPSSNKTVIATLSIEDSESGLSSRHHPTIYLQDVFGTLISISIGNGMTVVDDTVFTRTYSCIFT
IPFGFGYPAGASIAVYGIVDRFMNTRGYN**HYSLFYDIGR**GTVSPFISTSYSLAPSIVNMVPTFDPDYKSA
QFIIYGSFLGLVSSDVQLTIEYISESSQSSSQDSQSQEFTLSPRNILSTTTTTVPFTVSSIFGNLIAISV
NLQNAISRQIQIQLNVAGQPSNVYNFILPSEVIDSTSTSGISTTTTATTTTTTQDSTSGSATSGGGSTTT
GGGSSTTSTTTGTSGPDCSKLSNCGGSSQGNCVADNTCSCINSWIGVDCNSKPLKGVSINVNPNAPSASL
DQDDGSGVKLSSLINLYQIREIDSVTNKIIYQYDLQDKWVPVKQASPIPNTFIYSNTLSNAKSSVIYAKI
AIYDKQSTFEFAGQQYSINPNSIKFTINITNYKVDNVLNNLELIMQAQLNSSQTDDICSKKEVVKGSGSD
QLSVQLNNKSLYCRFLNSALLDGVTVVKNLTHVNLNSEFQALVEPNSGQYFYGIVLPTFNDYIILDPDFS
LLLQSSTISSDDGGVCSSSSSKKFPVG**AIVGIVVGGVAFIVVIAIVISTILF**RRHRFSSVTMKLRTWNSK
RRSKSKSYNLKNF

Sequenced Peptides, Signal Peptide, Transmembrane Domain, Antibody Epitope

Figure S3
